# Exclusive enteral nutrition mediates gut microbial and metabolic changes that are associated with remission in children with Crohn’s disease

**DOI:** 10.1038/s41598-020-75306-z

**Published:** 2020-11-03

**Authors:** Kay Diederen, Jia V. Li, Gillian E. Donachie, Tim G. de Meij, Dirk R. de Waart, Theodorus B. M. Hakvoort, Angelika Kindermann, Josef Wagner, Victoria Auyeung, Anje A. te Velde, Sigrid E. M. Heinsbroek, Marc A. Benninga, James Kinross, Alan W. Walker, Wouter J. de Jonge, Jurgen Seppen

**Affiliations:** 1 Department of Pediatric Gastroenterology and Nutrition, Amsterdam UMC, Location AMC & VUmc, Amsterdam, The Netherlands; 2 Tytgat Institute for Liver and Intestinal Research, Amsterdam UMC, Location AMC, Meibergdreef 69, 1105BK Amsterdam, The Netherlands; 3 Division of Digestive Diseases, Department of Metabolism, Digestion and Reproduction, Imperial College London, London, UK; 4 Department of Surgery and Cancer, Imperial College London, London, UK; 5 The Rowett Institute, University of Aberdeen, Aberdeen, UK; 6 Pathogen Genomics Group, Wellcome Sanger Institute, Hinxton, Cambridgeshire, UK

**Keywords:** Gastrointestinal diseases, Microbiology, Gastroenterology

## Abstract

A nutritional intervention, exclusive enteral nutrition (EEN) can induce remission in patients with pediatric Crohn’s disease (CD). We characterized changes in the fecal microbiota and metabolome to identify the mechanism of EEN. Feces of 43 children were collected prior, during and after EEN. Microbiota and metabolites were analyzed by 16S rRNA gene amplicon sequencing and NMR. Selected metabolites were evaluated in relevant model systems. Microbiota and metabolome of patients with CD and controls were different at all time points. Amino acids, primary bile salts, trimethylamine and cadaverine were elevated in patients with CD. Microbiota and metabolome differed between responders and non-responders prior to EEN. EEN decreased microbiota diversity and reduced amino acids, trimethylamine and cadaverine towards control levels. Patients with CD had reduced microbial metabolism of bile acids that partially normalized during EEN. Trimethylamine and cadaverine inhibited intestinal cell growth. TMA and cadaverine inhibited LPS-stimulated TNF-alpha and IL-6 secretion by primary human monocytes. A diet rich in free amino acids worsened inflammation in the DSS model of intestinal inflammation. Trimethylamine, cadaverine, bile salts and amino acids could play a role in the mechanism by which EEN induces remission. Prior to EEN, microbiota and metabolome are different between responders and non-responders.

## Introduction

Exclusive enteral nutrition (EEN) is the treatment of choice for patients with pediatric Crohn’s disease (CD) in Europe^1^. It is equally efficacious as corticosteroids in inducing clinical remission in children and adolescents with active CD^2,3^ and superior in inducing mucosal healing^4^. Despite solid evidence for efficacy of nutritional therapy in children, the mechanisms by which EEN induces remission remain elusive.

The gut microbiota and metabolomes of patients with CD are different from healthy individuals^5,6^. The implication is that perturbed gut microbial co-metabolism could play an important role in the pathogenesis of CD. Indeed many studies showed that interactions between the gut microbiota, metabolites and the intestinal immune system are critical for maintenance of a healthy intestine^7^. The intestinal microbiota of new-onset patients with pediatric CD typically exhibits a decreased microbial diversity^8^, with lower relative abundance of *Firmicutes*^9^. Moreover, patients with CD exhibit profound differences in the intestinal metabolome, including lower concentration of short chain fatty acids^10,11^, higher concentration of amino acids^11^, and a dysregulation of bile acid composition, including higher conjugated and lower secondary bile acid concentrations^12^.

EEN therapy likely modulates both the microbial and metabolic environments in the gut of patients with CD^13–15^. Several studies demonstrated that EEN reduces the alpha diversity of the intestinal microbiota of patients with CD^14,15^. This appears paradoxical, as higher alpha diversity is often associated with a more “healthy” microbiota. More specifically, EEN was found to induce a decline in numbers of presumably protective gut bacterial species (e.g. *Faecalibacterium prausnitzii* and *Bifidobacterium* spp*.*). In addition, decreased concentrations of fecal short chain fatty acids (SCFAs) such as butyrate, which is generally thought to be a beneficial metabolite for host health, have been found to be associated with disease improvement during EEN^15^. However, from a nutritional and microbiological point of view, these findings are to some extent expected. Because EEN contains relatively few components, when compared to a regular diet, a reduced alpha diversity of the gut microbiota is likely to result in people with this diet. In addition, removing complex carbohydrates from the diet reduces the amount of substrate available for fermentation into SCFAs by fiber-degrading bacterial taxa.

Whether changes in the intestinal microbiota and metabolome are a cause or consequence of CD remains uncertain, primarily due to the lack of longitudinal observations^8,16^. Two recent papers show that diets with either partial EEN^17^ or mimicking EEN composition with more solid ingredients^18^, are equally effective as EEN in inducing remission in patients with pediatric CD. These papers^17,18^ are important because the novel nutritional therapies are better tolerated and would thus be beneficial for a larger number of patients. Moreover, these papers^17,18^ also show that changes in the fecal microbiota and metabolome induced by these novel nutritional therapies are comparable to the changes that are induced by EEN. The findings reported thus also provide evidence that changes in the microbiota and metabolome could be causative in inducing remission in patients with pediatric CD disease.

In this study we prospectively followed a cohort of pediatric treatment-naïve patients with CD undergoing EEN therapy and investigated changes in their fecal microbiota and metabolome. The aims of this study were; (1) to describe gut microbial and metabolic changes during the course of EEN and the differences between responders and non-responders to EEN. (2) To evaluate these microbial and metabolic changes as potential mechanisms of EEN action.

## Results

### Patients

**Figure 1:**
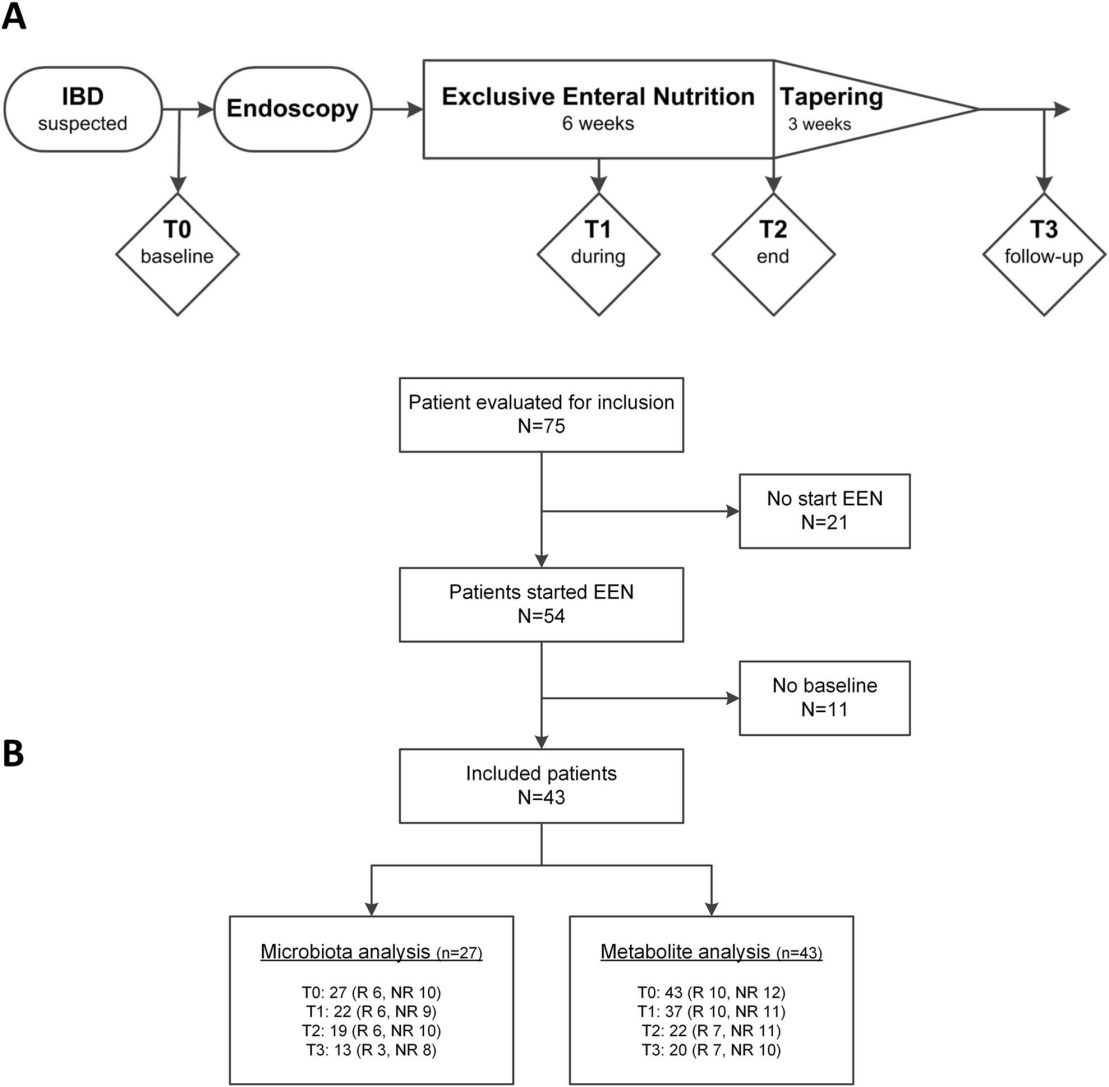
Study outline and patient selection. Panel (**A**) shows the therapy and time points of fecal sample collection. In panel (**B**) the selection of patients that were included in this study is outlined.

In total, 43 children with newly diagnosed CD were included (47% male, median age, 14 years [IQR 12–15], Fig. 1A,B). Patient and disease characteristics, and clinical and biochemical response are shown in Fig. 1B and Table 1 and were partially described previously^19^. In addition to EEN, all patients were started on concomitant thiopurines (i.e. azathioprine) following the international guidelines^1^. Eighteen healthy controls participated (50% male, median age years 13 [IQR 11–16]). For the microbiota analysis 96 samples were included, at baseline (T0) 27 samples, during EEN (T1) 22 samples, at end of EEN (T2) 19 samples, at follow up on habitual diet (T3) 13 samples, and 15 healthy control samples. For the metabolite analysis 122 samples were included, at baseline (T0) 43 samples, during EEN (T1) 37 samples, at end of EEN (T2) 22 samples, at habitual diet (T3) 20 samples, and 31 healthy control samples were included.

**Table 1:** Patient characteristics of included CD patients.

	Total Crohn’s disease patients (n = 43)	
	Microbiota analysis (n = 27)	Metabolite analysis (n = 43)
Age (median, IQR)	14 (12–15)	14 (12–15)
Males	14 (52%)	20 (47%)
**Crohn’s disease: age at diagnosis (Paris classification)**		
A1a: 0– < 10 years	3 (11%)	4 (9%)
A1b: 10– < 17 years	24 (89%)	39 (91%)
**Crohn’s disease: location**^**a**^** (Paris classification)**		
L1: distal 1/3 ileum	2 (7%)	5 (12%)
L2: colonic	2 (7%)	9 (21%)
L3: ileocolonic	23 (85%)	29 (67%)
Clinical responders	17 (63%)	28 (65%)
Biochemical responders that completed EEN	6 (38%) (total n = 16)	10 (45%) (total n = 22)

*CD* Crohn’s disease.

^a^L1: distal 1/3 ileum ± limited cecal disease; L2: colonic; L3: ileocolonic; L4a: upper disease proximal to the ligament of Treitz; L4b: upper disease distal to ligament of Treitz and proximal to distal 1/3 ileum.

Some patients halted EEN due to various reasons. The samples of these patients were analyzed up to the latest time point at which they did receive EEN and were excluded from further study.

Regarding control samples used in this study; two time points of the 18 control subjects were analyzed in the metabolic analysis. Five samples had to be excluded because of preparation errors. For the microbiome analysis only 15 controls at a single time point were used because of limitations in sequence capacity. The overall study design is shown in Fig. 1A,B.

Responders to EEN were identified by a reduction of fecal calprotectin of more than 50% at T2, compared to T0. See Fig. 1B and Table 1 for patient inclusions. Pilot analysis did not reveal statistically significant correlations when using the clinical definitions for remission (data not shown).

### Microbiota

#### Healthy controls versus patients with CD

To investigate the fecal microbiota of patients with CD and controls and the effect of EEN treatment, 16S rRNA gene amplicon sequence analysis was carried out.

**Figure 2:**
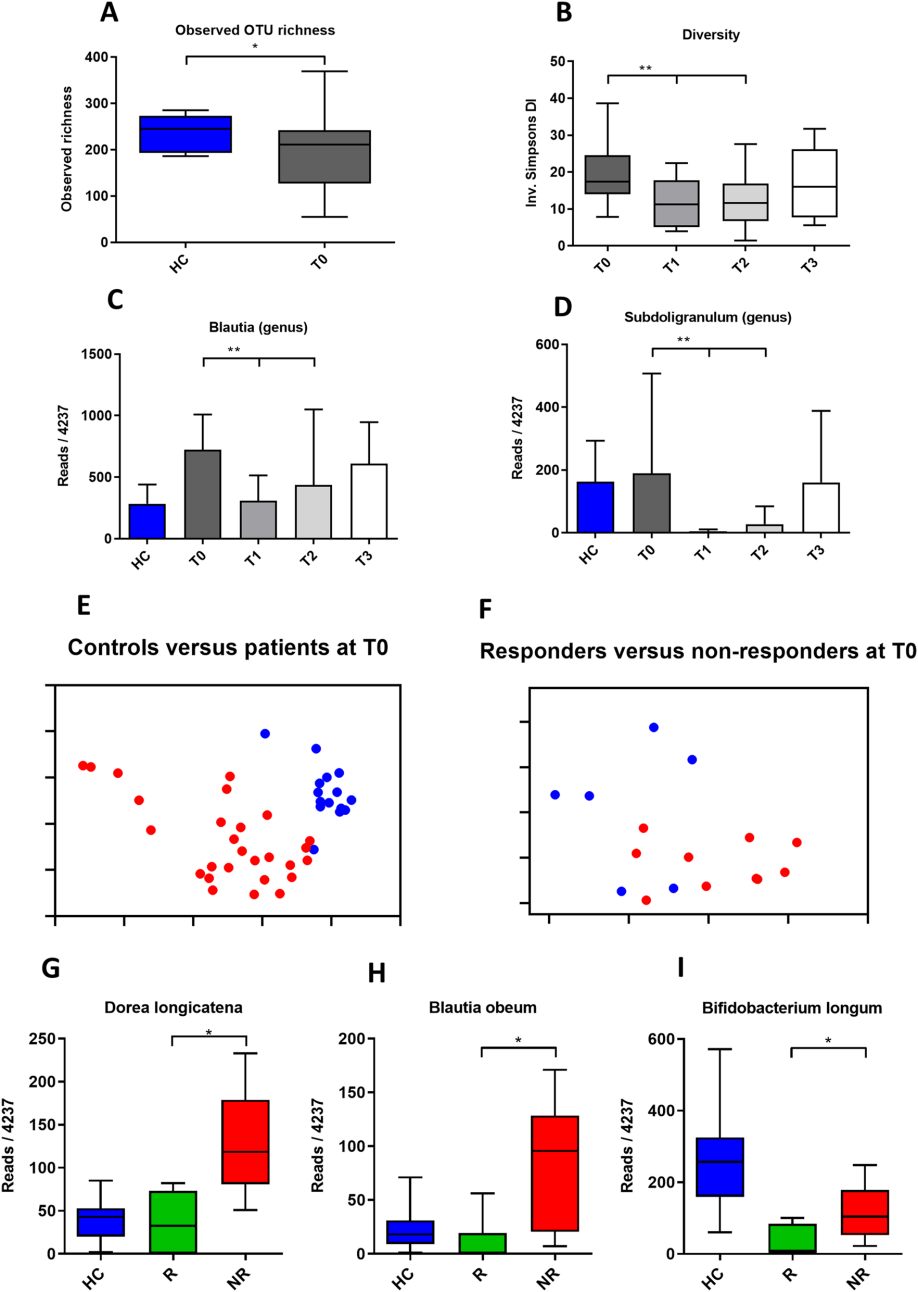
Microbiota of patients versus controls, the effect of EEN and differences between responders and non-responders. Panel (**A**) shows the difference in richness between controls and patients at T0. Panel (**B**) shows that EEN reduced diversity. Panel (**C**) and (**D**) show the effect of EEN on the proportional abundance of the *Blautia* and *Subdoligranulum* genera. Principal coordinate (PCoA) plots in panels (**E**) and (**F**) show separation of controls (red) and patients (blue), and responders (red) and non-responders (blue) at T0. In panels (**G**,**H**) and (**I**) some of the species that comprise the signature of responders versus non-responders at T0 are depicted. *HC* healthy controls, *R* responders, *NR* non-responders.

At baseline, patients had reduced observed OTU richness, as compared to healthy controls (HC, mean OTU richness of 235.6 vs. CD T0, mean OTU richness of 192.9, *p* = 0.036, Mann–Whitney, Fig. 2A). Shannon and inverse Simpson diversity measures, which incorporate species evenness as well as richness, were not significantly different between controls and patients at baseline (HC 3.67 vs. T0 3.4, *p* = 0.098, and HC 20.37 vs. CD T0 16.75, *p* = 0.198, respectively).

As expected, the overall microbial composition of controls was highly different (*p* < 0.001) from that of patients at all time points (Bray Curtis with AMOVA, Table 2, Fig. 2E). Numerous taxa exhibited significantly different proportional abundances; *Escherichia coli*, *Ruminococcus gnavus*, *Dorea longicatena*, and *Blautia* spp. were present in greater relative abundance in CD, whereas *Eubacterium rectale*, *Bifidobacterium longum* and *Ruminococcus bromii* were proportionally higher in controls (all *p* values < 0.05 with both LEfSe and Benjamini–Hochberg-corrected *p* values generated by Metastats) Supplementary Table 4 details the differences of the most proportionally abundant species between patients with CD at T0 and controls.

**Table 2:** Differences in overall bacterial community composition between controls (HC) and patients at T0, and between patients during EEN.

	*p*
HC versus T0	< 0.001*
HC versus T1	< 0.001*
HC versus T2	< 0.001*
HC versus T3	< 0.001*
T0 versus T1	0.001*
T0 versus T2	0.001*
T0 versus T3	0.9999
T1 versus T2	1.000
T1 versus T3	0.025*
T2 versus T3	0.093

Controls were different from patients at all time points. EEN induces significant changes in microbial composition, T0 versus T1 and T2. At follow up T3, microbial composition of patients was not different from that at the start, T0.

Differences in community composition were analyzed using the AMOVA test in mothur, using Bray Curtis dissimilarity measures.

#### The effect of EEN on the microbiota

EEN therapy caused a decrease in microbial diversity, with patients at T1 and T2 having a significantly lower diversity than at baseline (mean inverse Simpson Index score for T0 = 19.40 vs. T1 = 11.57, *p* = 0.003) (T0 = 19.40 vs. T2 = 12.85, *p* = 0.045) (Fig. 2B). At follow up (T3) there was no difference in diversity from baseline (mean T0 inverse Simpson index score = 19.40 vs. T3 mean = 16.87, *p* = 0.505).

The introduction of EEN was also significantly associated with a shift in the overall microbial composition (T0 vs. T1, *p* =  < 0.001) and at the end of EEN (T0 vs. T2, *p* =  < 0.001), with differences disappearing at follow up (T0 vs. T3, *p* = 0.99) (Bray Curtis-based AMOVA tests, Table 2). However, the response of individual taxa varied greatly between individuals. (Supplementary Figure 1). Only genera that were identified as being significantly different using the stringent, Benjamini–Hochberg-corrected, Metastats-based approach, and with a proportional abundance of more than 0.01% of the total observed microbiota, are detailed below. The genera significantly altered between baseline (T0) and during the EEN treatment (T1) were *Blautia* (10.8% of the total observed microbiota across all samples) and *Subdoligranulum* (2.5% of the total observed microbiota), both of which were reduced in relative abundance during EEN therapy, (Metastats-based *p* = 0.033 and *p* = 0.044, respectively) (Fig. 2C,D). However, neither of these genera was significantly reduced at timepoint T2 compared to T0. No bacterial taxa were shown to be significantly increased in proportional abundance with both the Metastats-based and LEfSe approaches as a result of the EEN therapy.

#### Responders versus non-responders

Prior to the onset of EEN therapy, the overall composition of the microbiota differed between subsequent responders and non-responders (Bray Curtis dissimilarity analysis and AMOVA tests, *p* = 0.008). A PCoA plot is shown in Fig. 2F.

Due to the high inter-individual variation in microbiota composition, there were no significant differences in alpha diversity measures, and few species or genera that differed between responders and non-responders prior to therapy (T0). However, *Dorea longicatena* (2.5% of the total observed microbiota) (Metastats-based *p* = 0.012; LEfSe *p* = 0.005), (Fig. 2G) *Blautia obeum* (1.3% of the total observed microbiota) (Metastats-based *p* = 0.019; LEfSe *p* = 0.006) (Fig. 2H), and *Bifidobacterium longum* (0.07% of the total observed microbiota) (Metastats-based *p* = 0.040; LEfSe *p* = 0.012) (Fig. 2I) were all associated with lack of response. Using three-way LEfSe analysis, comparing healthy controls versus responders (T0) versus non-responders (T0), multiple taxa appeared to be associated with both responders and non-responders at T0. Supplementary Table 5a details the most important differences between healthy controls, responders and non-responders at T0. The following taxa are examples of proportionally abundant components of the bacterial “signature” that differentiated non-responders from responders prior to therapy (T0): *Dorea longicatena* (Non-responders, *p* =  < 0.001), *Blautia obeum* (Non-responders, *p* = 0.002), and *Escherichia coli* (Non-responders, *p* =  < 0.001).

At the follow up period (T3), there were also differences between responders and non-responders. As with T0, the overall composition of the microbiota at T3 also differed between responders and non-responders (Bray Curtis dissimilarity analysis and AMOVA tests, *p* = 0.004). (PCoA plot shown in Supplementary Figure 3A). Of note, both Metastats-based and LEfSe analyses associated higher proportional abundances of the butyrate-producing genus *Roseburia* with responders (2.40% mean proportional abundance at T3 in responders vs. mean of 0.22% in non-responders, Benjamini–Hochberg-corrected Metastats-based *p* = 0.043, LEfSe *p* = 0.013), while another key butyrate-producing genus, *Faecalibacterium*, was also associated with responders by LEfSe only (10.48% mean proportional abundance in responders vs. mean of 1.42% in non-responders, *p* = 0.041). Three-way LEfSe analysis, comparing healthy controls versus responders (T3) versus non-responders (T3), associated, amongst others, *Faecalibacterium prausnitzii* (*p* = 0.034), *Bifidobacterium adolescentis* (*p* = 0.032), and *Ruminococcus bromii* (*p* = 0.009) with responders, and *Escherichia coli* (*p* = 0.004) and four *Blautia* OTUs (*p* = 0.003, 0.008, 0.026, and 0.009) with non-responders.

Supplementary Table 5b details the most important differences between healthy controls, responders and non-responders at T0 and at T3.

Thus, EEN significantly changes both alpha and beta diversity indices in patients with CD, and we provide evidence for differences between responders and non-responders both prior to and after therapy.

### ^1^H NMR spectroscopy-based metabolic profiling analysis

#### Healthy controls versus patients with CD

Metabolic differences between patients with CD and controls and the effects of EEN were analyzed using ^1^H NMR spectroscopy.

**Figure 3:**
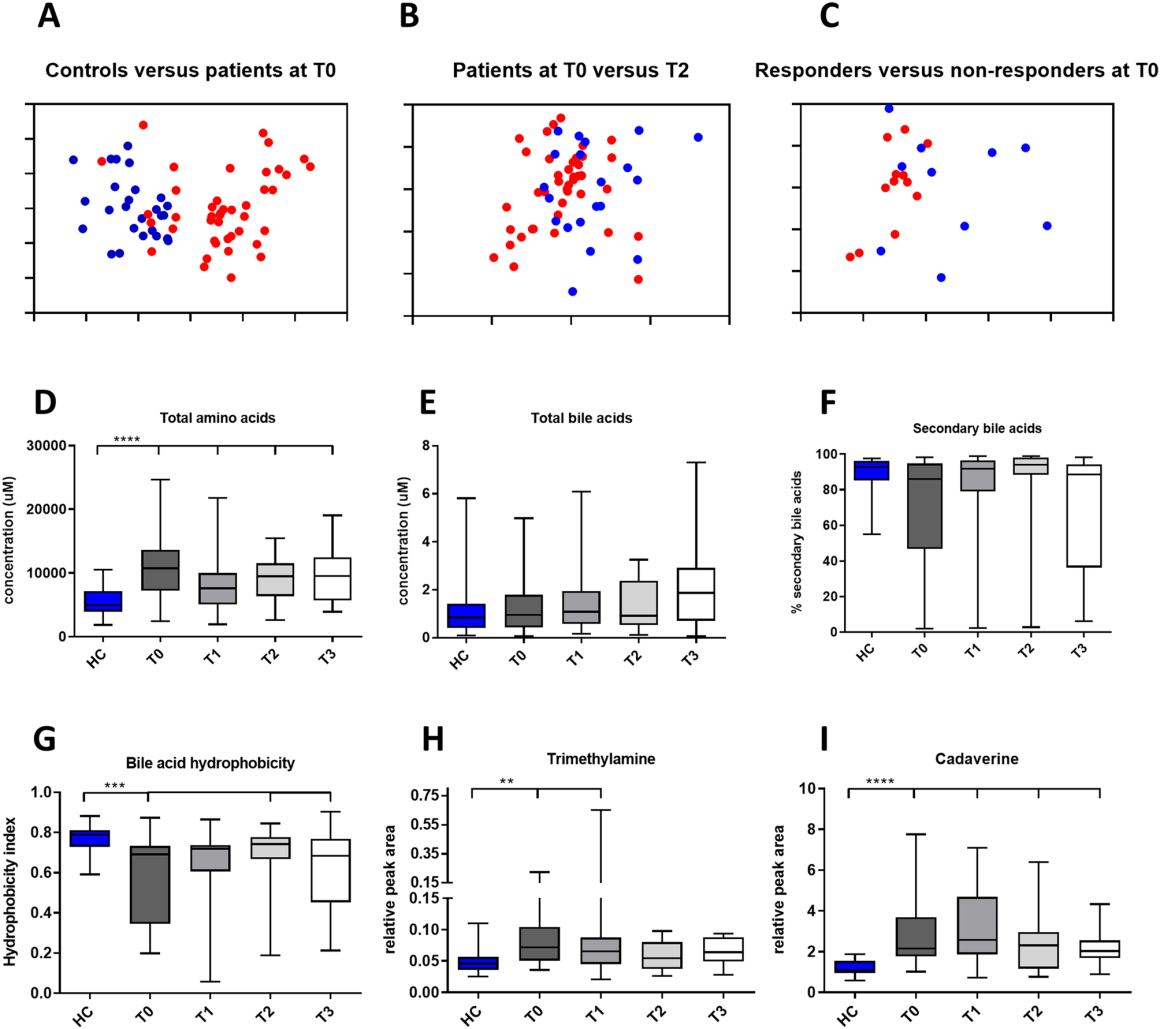
Differences in fecal metabolites between patients and controls, responders and non-responders and the effect of EEN on the fecal metabolome. Panels (**A**–**C**) show OPLS-DA plots. This analysis indicates that ^1^HNMR analysis can distinguish controls (blue) from patients (red) at T0, panel (**A**). A marginal EEN induced change in metabolome from T0 (red) to T2 (blue) is shown in panel (**B**). The difference in metabolome between responders (blue) and non-responders (red) at T0 is shown in panel (**C**). Panel (**D**) shows increased total fecal amino acids in patients that decrease during EEN therapy. Panel (**E**) shows that bile salts are not elevated in patients and unchanged during therapy. Panels (**F**) and (**G**) show decreased bile salt metabolism and hydrophobicity in patients at T0 that is partially normalized during therapy. Panels (**H**) and (**I**) show increased levels of trimethylamine and cadaverine respectively and show partial normalization of these compounds during EEN therapy. *HC* healthy controls, *R* responders, *NR* non-responders.

Significant differences were observed between patients with CD and HC at all time points based on Orthogonal Partial Least Squares-Discriminant Analysis (OPLS-DA) models. An example of the cross-validated scores plot of OPLS-DA between the HC and patients with CD at T0 is shown in Fig. 3A (R^2^X = 59.7%, Q^2^Y = 0.55, permutation *p* = 0.01). The corresponding ROC curve (Supplementary Figure 2A, AUC = 0.94) confirms that this model is highly predictive to distinguish patients from controls. Amino acids (alanine, tryptophan, tyrosine, valine, isoleucine, leucine, phenylalanine) and microbial metabolites (cadaverine, lactate, propionate, putrescine, trimethylamine (TMA)) were found in higher concentrations in patients with CD at T0 compared to healthy controls (Supplementary Table 6).

#### The effect of EEN on the metabolome

EEN affected the global metabolome with the fecal metabolic profile prior to EEN (T0) differing significantly from the end of EEN (T2) (R^2^X = 59.4%, Q^2^Y = 0.1, *p* = 0.04, Fig. 3B). However, the corresponding ROC curve (Supplementary Figure 2B, AUC 0.71) does not suggest a highly predictive value of the model at this time point. No significant differences were found between other time points.

#### Responders versus non-responders

When patients were stratified based on their responses to EEN therapy, there was a statistically significant difference between the fecal metabolic profiles of responders and non-responders at baseline (T0) (R^2^ = 60.4%, Q^2^ = 0.28, *p* = 0.030, Fig. 3C). The ROC curve (Supplementary Figure 2C, AUC = 0.8) suggests that the fecal metabolic profiles could predict the responses of patients to EEN prior to the treatment.

Although the global metabolic profiles of both responders (R^2^X = 58.0%, Q^2^Y = 0.33, *p* = 0.01) and non-responders (R^2^X = 67.5%, Q^2^Y = 0.69, *p* = 0.01) were different from healthy controls at follow-up (T3), several metabolites (i.e. leucine, propionate, valine, lactate, alanine, cadaverine, trimethylamine, tyrosine, phenylalanine, isovalerate, urocanate, succinate) were normalized in responders but not in non-responders (Supplementary Table 6).

### Amino acids

#### Healthy controls versus patients with CD

Because the NMR analysis showed large differences in amino acids between patients with CD and controls, we investigated fecal amino acids in more detail using HPLC. At baseline (T0), there was a clear separation of the fecal amino acid profile in CD from HC, with a higher concentration of the total and most individual amino acids (Fig. 3D, Supplementary Table 7, Supplementary Table 8). Only glutamic acid, arginine and taurine were not elevated in patients.

#### The effect of EEN on amino acids

Total amino acid concentrations tended to decrease at T1 when compared to T0. However, the fecal amino acid profiles at baseline (T0) were not significantly different from time-points (T1, T2 & T3), for either the total or individual amino acid concentrations (Fig. 3D, Supplementary Table 7, Supplementary Table 8).

#### Responders versus non-responders

When patients were stratified based on response to EEN therapy, responders had a lower fecal concentration of histidine, citrulline, and isoleucine both at baseline (T0) and at the end of EEN therapy (T2), and a lower concentration of serine, glycine, and alanine at end of EEN therapy (T2) (Supplementary Table 9).

We also compared amino acids between healthy controls, responders and non-responders. Although some individual amino acids of both responders and non-responders had a higher concentration in healthy controls at follow-up (T3) (i.e. asparagine, tryptophan), many amino acid concentrations were normalized in responders and not in non-responders at T3 (i.e. serine, histidine, tyrosine, phenylalanine, leucine), (Supplementary Table 8).

#### CD does not affect systemic amino acids

To establish whether the elevated fecal amino acids in patients with CD are reflected in the systemic circulation we measured plasma amino acids in an independent cohort of pediatric IBD patients (N = 41, 63% male, median age 15 years [IQR 12–16], median disease duration 22 months [IQR 10–44], 76% CD, 24% ulcerative colitis). No correlation was found between plasma total, or individual amino acids concentrations and disease severity as measured by fecal calprotectin (r: 0.166, *p* = 0.306, Supplementary Figure 3B).

### Bile acids

#### Healthy controls versus patients with CD

Bile salts are emerging as important mediators in immune function^20^. Because little is known about bile salt metabolism in CD and during EEN, we analyzed the fecal bile acid composition by HPLC. Although the total concentrations of bile acids were not different between patients with CD and controls (Fig. 3E, Supplementary Table 10), the metabolism of fecal bile acids was significantly changed in patients with CD (Fig. 3F, Supplementary Figure 3D–H). At baseline (T0), the relative concentration of primary bile acids was higher in patients with CD resulting in a reduced hydrophobicity of the bile acid pool (Fig. 3G, Supplementary Table 10).

#### The effect of EEN on bile acids

Comparing the fecal bile acids at baseline (T0) to during EEN (T1), or time-points after EEN cessation (T2 & T3), revealed no difference in bile acids hydrophobicity or the percentage of secondary bile acids (Fig. 3G, Supplementary Table 11).

#### Responders versus non-responders

When patients were stratified based on response to EEN therapy, there was no difference in the total bile acid concentration, bile acid hydrophobicity and the fraction of secondary bile acids between responders and non-responders at baseline (T0), or other time points (T1-3) (Supplementary Tables 10 and 11).

At follow-up (T3), the fraction of secondary bile acids and the total bile acid concentration in feces of responders and non-responders did not differ from HC (Supplementary Table 10). However, bile acid hydrophobicity was normalized in responders and not in non-responders at follow-up (T3) (Supplementary Table 10).

### Effects of selected metabolites on physiological parameters

After establishing the effect of EEN on fecal metabolic composition we investigated if metabolites that were shown to be different between controls and patients with CD could have a causal role in disease development.

#### TMA and cadaverine inhibit Caco-2 proliferation

Since wound healing is a critical process in recovery from inflammation we used a real time assay of cell growth and attachment to study the effect of selected metabolites on this process.

TMA and cadaverine were metabolites of interest as they differed between patients with CD and healthy controls and are metabolic products of intestinal microbes (Fig. 3H,I)^21,22,23^. These compounds were especially interesting because they were normalized in responders only at follow up (Supplementary Table 6). As a control metabolite for TMA we selected TMA *N*-oxide (TMAO), which is produced from TMA by hepatic flavin monooxygenases 3 (FMO3)^24^.

**Figure 4:**
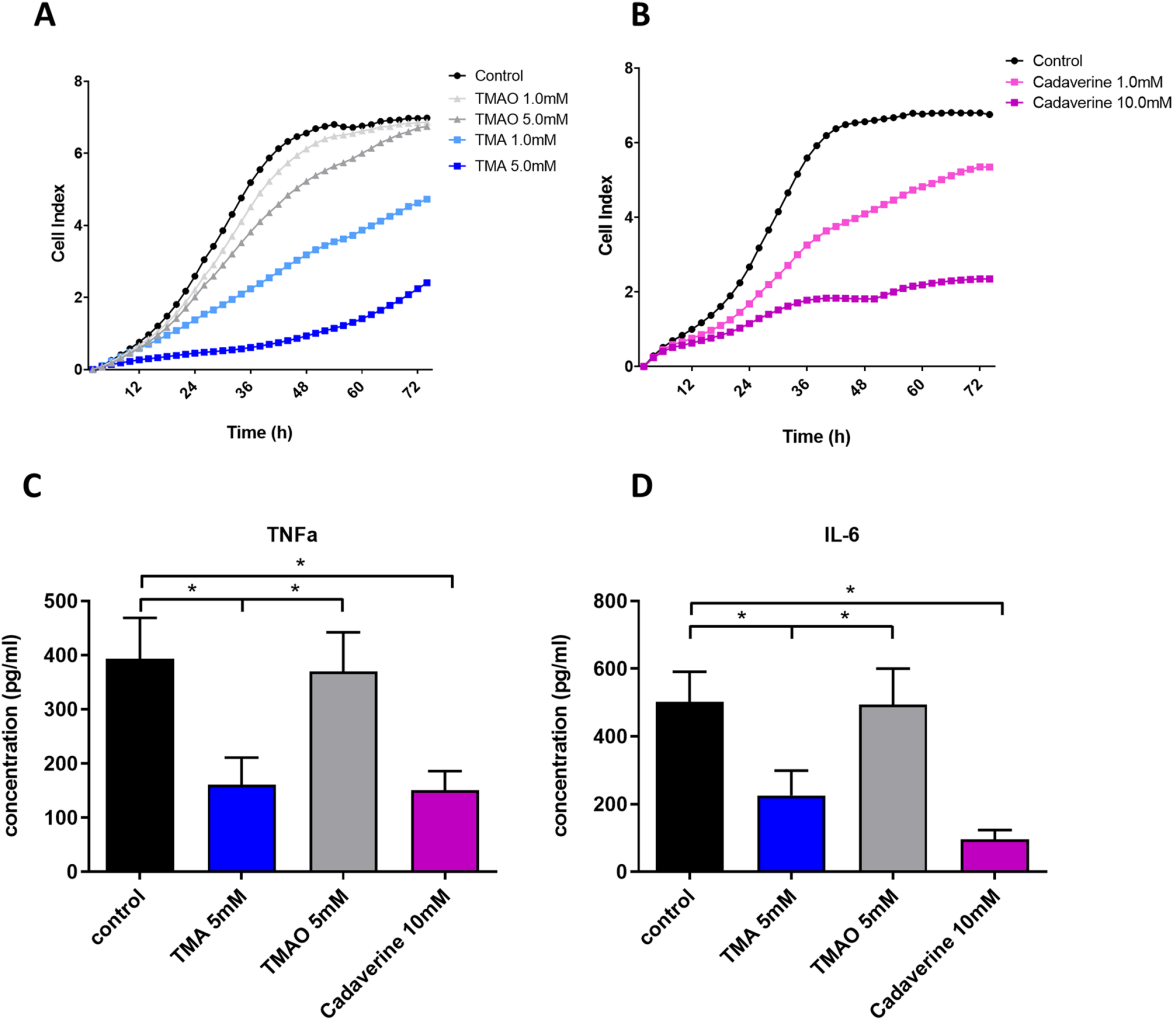
The effect of TMA and cadaverine on cell growth, differentiation and cytokine secretion. Panel (**A**) shows that TMA, but not its metabolite TMAO reduces CaCo_2_ cell growth in a concentration dependent manner. Panel (**B**) shows that cadaverine reduced CaCo_2_ cell growth in a concentration dependent manner. Panels (**C**) and (**D**) show that TMA, but not its metabolite TMAO, and cadaverine are both able to reduce LPS induced TNFα and IL-β secretion in primary human PBMC.

Both TMA and cadaverine inhibited caco-2 cell proliferation, relative to standard DMEM medium (Fig. 4A,B, Table 3). The control metabolite TMAO, did not inhibit caco-2 cell proliferation (Fig. 4A, Table 3).

**Table 3:** The effect of the metabolites TMA and cadaverine on epithelial cell proliferation and adhesion (cell index).

	*p*: 24 h	*p*: 48 h	*p*: 72 h
**TMA versus control**			
TMA 1.0 mM versus control (DMEM)	< 0.001	< 0.001	0.021
TMA 5.0 mM versus control (DMEM)	< 0.001	< 0.001	< 0.001
**TMA versus TMAO**			
TMA 1.0 mM versus TMAO 1.0 mM	0.001	0.004	0.010
TMA 5.0 mM versus TMAO 5.0 mM	< 0.001	< 0.001	< 0.001
**Cadaverine versus control**			
Cadaverine 1.0 mM versus control (DMEM)	< 0.001	< 0.001	< 0.001
Cadaverine 10.0 mM versus control (DMEM)	< 0.001	< 0.001	< 0.001

Cell index was measured on CaCo_2_ cells using a real time assay. TMA and cadaverine significantly inhibited proliferation and adhesion at all concentrations tested compared to controls.

Cell indices were compared using the Mann–Whitney U test.

#### TMA and cadaverine reduce LPS induced TNFα and IL-6 secretion in primary human lymphocytes

A possible mechanism by which microbial metabolites induce remission is by modulating the host immune response. We therefore studied the effect of selected metabolites on LPS induced cytokine secretion in primary human lymphocytes.

Human peripheral blood monocytes were incubated with TMA, TMAO and cadaverine for 24 h and subsequently stimulated with LPS. IL-6 and TNFα release from primary human monocytes upon LPS stimulation was inhibited by TMA and cadaverine but not by TMAO (Fig. 4C,D), *p* < 0.0001. The differences in IL-6 and TNFα release by monocytes were not caused by increased cell death as shown by FACS viability staining (Supplementary Figure 3C).

#### Amino acid feeding worsens outcome of a mouse model of intestinal inflammation

Since amino acids were elevated in patients, and many amino acids were normalized in responders at T3 only (Supplementary Table 9) we wanted to investigate a causal role of these compounds in inflammation. As cell culture models are not suitable for studying the effect of amino acids, we determined if these compounds could affect the DSS model of intestinal inflammation.

**Figure 5:**
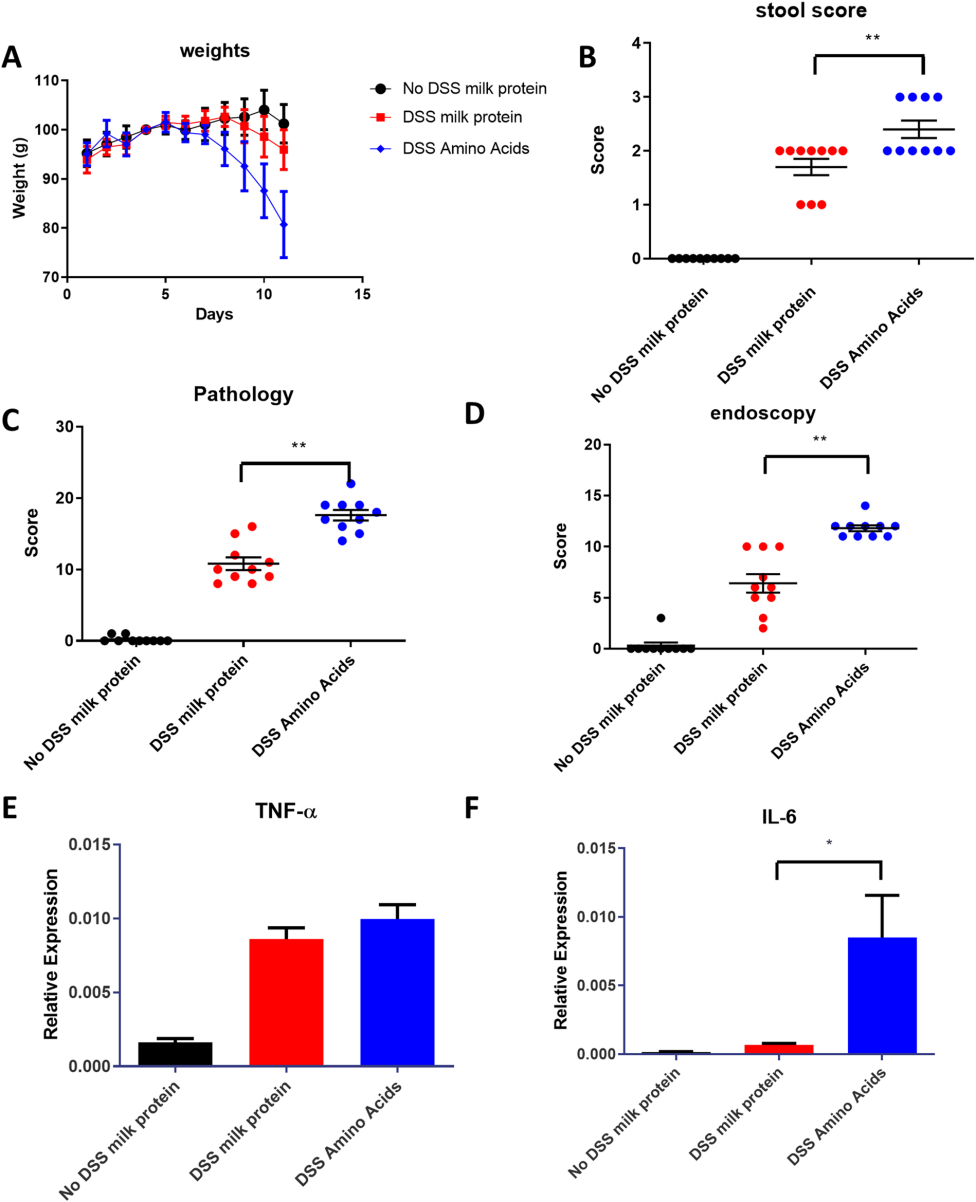
A diet of amino acids worsens the murine DSS model of colitis. Mice received 1.5% DSS in their drinking water and were fed chow supplemented with milk protein (DSS milk protein) or an equivalent amount of amino acids (DSS Amino Acids). Control mice received milk protein (No DSS milk protein). Mice fed amino acids lost weight more rapidly (panel **A**), had increased stool, pathology and endoscopy scores, (panels **B**–**D**). Levels of TNFα, as measured by quantitative PCR, were elevated in both groups (panel **E**) whereas levels of IL-6, as measured by quantitative PCR, were only elevated in the group receiving amino acids (panels **F**).

Mice were fed amino acids or a diet containing a caloric equivalent amount of whole milk protein. Non-DSS treated mice fed with standard chow served as a control group. Weight loss, stool score, pathology, and endoscopy score were all worse in DSS treated mice (both amino acid or milk protein fed) compared to the control group (Fig. 5A–D). Amino acid-fed DSS mice had a lower body weight, and significantly (*p* < 0.0001) worse pathology and endoscopy score compared to milk-protein fed DSS mice (Fig. 5A–D).

TNF-α expression was higher in the colons of DSS treated mice (both amino acid or milk protein fed) compared to control mice (Fig. 5E), IL-6 was only higher in the colons of DSS-treated mice fed with amino acids (Fig. 5F). Amino acid-fed mice showed a significantly (*p* = 0.028) increased expression of IL-6 over those fed with milk protein (Fig. 5F). TNF-α, did not show differences between amino acid and milk protein fed mice (Fig. 5E).

## Discussion

In this study, we report a comprehensive analysis of the fecal microbiota and metabolome during a course of EEN therapy in patients with CD.

### Microbiota analysis

We found marked differences in microbiota between patients with CD and controls. The differences, as seen in this cohort, largely correspond to previous studies^25–28^.

The microbiota of children with CD was highly variable, as has been reported before^13^. Nonetheless, patients with CD had a reduced richness as compared to controls prior to therapy. In common with many studies^29,30^, we also detected increased proportional representation of *Escherichia coli* and *Ruminococcus gnavus*^31^ in patients with CD, and a reduction in the relative abundance of putatively beneficial organisms such as *Bifidobacterium* spp., and butyrate producers such as *E. rectale*. During EEN, diversity (as measured using the inverse Simpson Index) was further reduced, confirming results from a previous study^14^.

Although limited by our relatively small number of samples, our results suggest that increased proportional abundance of taxa such as *Bifidobacterium longum*, *Dorea longicatena*, and *Blautia obeum* may indicate a lower chance of disease remission following EEN. Our observations suggest that the *Blautia* genus is of particular interest since proportional abundances of this group were generally higher in patients with CD versus healthy controls at baseline, were reduced in patients with CD during the EEN period, and were associated with non-responders at both T0 and T3. In contrast, a randomized clinical trial comparing nutritional interventions in pediatric patients with CD reported an increase in the *Blautia* genus during EEN^17^.

A meta-analysis of microbiota profiling studies in adult IBD patients reported an association of increased proportional abundance of *Blautia* spp. in patients with CD^32^. A more recent study showed that *Blautia* spp were associated with a dysbiotic cluster that separated part of patients with pediatric CD from controls^33^ Another study in pediatric CD reported increased *Blautia*, comparable to controls in patients with sustained response to Infliximab treatment^34^. Observations on *Blautia* *spp* from individual studies of pediatric CD cohorts thus appear to be variable, possibly because the resolution of genus level comparisons is not sufficient and masking contributions of *Blautia* species, or because the inherent inter-individual variation in microbiota composition makes it more difficult to find reproducible findings across different cohorts. More mechanistic research is evidently required in future in order to determine whether or not these species are playing a role in the disease process.

### Metabolites

In contrast to the microbiota, the metabolome trended towards normalization for many compounds as a result of EEN therapy. These observations are not completely surprising as the diversity measures used in this study reflects microbiota composition and not its metabolic activity.

Several microbial metabolites that could play a role in the pathogenesis of CD were identified in this study. Cadaverine and trimethylamine (TMA) were found in a higher concentration in patients with CD, tended to decrease during the course of EEN (Fig. 3H,I) and were normalized at T3 in responders only.

Increased amino acids have been previously observed in CD patients^35–37^. The most straightforward explanation for this phenomenon is a reduced uptake due to a damaged epithelium. However, because total bile salt concentrations were not different between patients with CD and controls, and glutamic acid and taurine levels were not elevated in patients with CD, reduced epithelial uptake capacity does not seem a likely explanation for increased amino acid levels.

### Mechanism of EEN

A possible causal role of elevated amino acids in CD is suggested by our observations that a diet containing amino acids worsens DSS induced colitis in a mouse model. These results are surprising in the light of previous studies showing that supplementation of amino acids reduces DSS-induced colitis^38,39^**.**

As examples, supplementation of mice in de DSS model of colitis with dietary tryptophan^40^, glutamine^41^, serine^42^ or arginine^43^ reduced inflammation.

However, study designs are not completely comparable, since we fed mice a complete mixture of amino acids compared to supplementation with selected amino acids. A recent paper shows that elevated fecal amino acids in patients with CD depended on increased urease activity of the microbiota^37^. Inoculation of a murine colitis model with *E. coli* engineered to express urease led to a worsening of the colitis in these animals. Increased amounts of amino acids seen in patients with CD might thus have a role in pathogenesis.

Cadaverine and TMA inhibit epithelial growth and adherence. This indicates that high levels of TMA and cadaverine could be detrimental for gut homeostasis and that these metabolites may be important in CD pathogenesis. Decreased serum levels of TMAO have been observed in patients with inflammatory bowel disease, providing additional evidence that reduced TMA metabolism might play a role in CD pathogenesis^44^.

Perhaps surprisingly, TNFα and IL-6 production of LPS stimulated human monocytes was reduced by TMA and cadaverine. However, besides its role as a pro-inflammatory cytokine, multiple anti-inflammatory effects of TNFα have also been described^45–47^.

Our data from the DSS model of colitis also suggest a complex role of TNFα and IL-6 in intestinal inflammation. Whereas amino acid feeding increased severity of intestinal inflammation, as assessed by multiple parameters, it only increased IL-6 levels, TNFα levels were unchanged.

Short chain fatty acids (SCFA) have been extensively studied in patients with IBD^48^. In this cohort, propionate concentrations were higher in children with CD, while butyrate levels were unaltered (not shown). Data regarding the effect of EEN on SCFA levels are conflicting, with decreases and increases both observed^15,49^.

The fecal bile acid pool of patients with CD was characterized by a higher concentration of primary and conjugated bile acids and lower secondary bile acids. This likely results in a fecal bile salt pool that is less hydrophobic in patients with CD. During EEN therapy, this alteration in bile acid composition was partially restored (Supplementary Figures 3D–H). Our results expand on an earlier study^12^, that showed similar alterations of the CD gut bile acids.

The anti-inflammatory effects of the TGR5 bile acid receptor activation are well described^48^. Since hydrophobic bile acids such as lithocholic acid are more potent activators of TGR5 than the hydrophilic bile acids^50^, the hydrophilic bile salt pool of patients with CD could be an important factor in the inflammatory process.

### Differentiating responders and non-responders prior to therapy

Fecal microbiota and metabolome of responders and non-responders differ before the start of EEN therapy. Microbial differences between responders and non-responders to immunomodulators or nutrition have been reported before^51,52^. This could indicate that there might be as yet unidentified subtypes of CD disease and open the possibility to identify non-responders before treatment. This approach seems feasible as a recent paper^53^ identifies microbial signatures that could predict long term responders to EEN.

### Conclusions

Patients responding to EEN therapy have different microbiota and metabolomes prior to therapy than patients that do not respond. This may allow for future prediction of EEN response.

The mechanism by which EEN induces remission is complex, several metabolites (TMA, cadaverine, amino acids and bile salts) possibly have a causal role in the development of CD.

## Patients, materials and methods

### Ethics

The Medical Ethical Committee of the Amsterdam UMC (Medisch Ethische Toetsingscommissie) approved the analysis of human samples described in this study under NL39254.029.12. In accordance with this approval, the children and their parents both gave informed consent for the use of pediatric samples in this study. All analysis and further experiments in this study were performed according to this approved protocol and relevant regulations and guidelines.

Animal experiments described in this study were performed according to a protocol that was approved by the Animal Ethical Committee of the Amsterdam UMC, (Dierexperimentele commissie) under nr DMO65. All analysis and further experiments in this study were performed according to this approved protocol and relevant regulations and guidelines.

### Patients

This was a prospective multi-center cohort study in two academic hospitals (Amsterdam UMC locations AMC and VUMC) performed between January 2010 and July 2014. All children (< 18 years) with newly diagnosed, therapy naïve CD according to the revised Porto criteria^54^ undergoing EEN induction treatment were included. The control group consisted of age and sex matched, healthy school children from the same geographic area, with no family history of inflammatory bowel disease (IBD), from a well-documented cohort^55^. An outline of analysis performed in this study can be found in Fig. 1A,B). Participants who received antibiotics or probiotics within 3 months prior to inclusion or during the study period were excluded. Moreover, participants with a proven bacterial gastroenteritis or who had taken immunomodulatory drugs 3 months prior to inclusion were also excluded. A range of polymeric EEN formulas with similar composition based on cow milk protein (Supplementary Table 1) was provided during a 6-week course, during which no other food or fluid (except water) was allowed, followed by a 2-week course of EEN tapering and gradual introduction of habitual diet. During follow-up, dose adjustments or switch of maintenance therapy (and reasons for therapy adjustment) were collected. Localization and disease behavior were classified using the Paris classification^56^.

### Sample collection

Patient and healthy controls were instructed to collect fecal samples in provided sterile containers, to store the sample at − 20 °C within at least 2 h of collection, and to deliver these frozen samples in a cooled condition to the hospital. A maximum of 4 samples were collected per patient at the following time points: At the time of diagnosis, but prior to bowel preparation and endoscopy (T0), during EEN (± 21 days after EEN initiation) (T1), at the end of treatment (± 42 days after EEN initiation) (T2), and after patients returned to their habitual diet (± 4 months after EEN initiation) (T3) (Fig. 1A). To avoid heterogeneity, samples from patients who had discontinued EEN therapy prematurely were excluded: T1 samples were excluded if EEN was previously discontinued, T2 and T3 samples were excluded if the full course of 6 weeks EEN had not been completed. Healthy controls were instructed to collect 2 fecal samples with an interval of 6 weeks. Aliquots of fecal samples were stored at − 20 °C until analysis.

### Biochemical and clinical disease activity

Biochemical disease activity was assessed using fecal calprotectin (FC), the most accurate fecal biomarker of intestinal inflammation currently available^57^. Fecal calprotectin levels were determined at baseline (T0) and end of EEN (T2) by the Amsterdam University Medical Centre hospital clinical chemistry laboratory. Biochemical response was defined as a reduction of ≥ 50% at T2 compared to T0, as this has the highest predictive value for endoscopic treatment response^58^. Because we did not obtain calprotectin from all patients and not all patients completed EEN therapy, for the analysis of responders versus non-responders not all samples could be used.

Clinical disease activity was assessed by the treating physician at T0 and T2 using the Physician Global Assessment (PGA) 4 point scale: inactive, mild, moderate, and severe^59^. Clinical response on EEN was defined as a PGA scored as inactive to mild after a full 6 week-course of EEN, without the need for additional remission induction treatment.

### Microbiota analysis using 16S rRNA gene amplicon sequencing

Sequencing of bacterial 16S rRNA gene amplicons was performed to characterize microbial community composition, largely as described in^60^, of which a modified version is described below.

#### DNA extraction

DNA extraction was carried out on fecal samples, ranging from 25 to 265 mg in weight, using the FastDNA SPIN Kit for Soil (116560200, MP Biomedicals) per kit instructions. Samples were eluted in 50 µl of DES then quality checked by running on an agarose gel.

#### PCR amplification for sequencing

PCR amplification of the V1-V2 hypervariable regions of the 16S rRNA gene was carried out using primers adapted for Illumina MiSeq, 27F (AATGATACGGCGACCACCGAGATCTAC-ACTATGGTAATTCCAGGTTYGATYMTGGCTCAG) and 338R individually barcoded (CAAGCAGAAGACGGCATACGAGAT-barcode-AGTCAGTCAGAAGCTGCCTCCCGTAGGAGT) primers. 1 µl of extracted DNA was run in a 25 µl PCR reaction containing 1.25 µl of each primer (10 µM), 5 µl of 5X Q5 Reaction Buffer, 0.25 µl of Q5 High-Fidelity DNA Polymerase (M0491, New England Biolabs), 0.5 µl of 10 mM dNTPs (N0447, New England Biolabs) and 15.75 µl of Nuclease-Free water as per Q5 standard protocol. Four PCR reactions were run for each sample using thermal cycler conditions of 98 °C for 2 min, then 20 cycles of 98 °C for 30 secs, 50 °C for 30 secs, 72 °C for 90 secs before a final extension of 72 °C for 5 min. Sample reactions were pooled and checked by running on an agarose gel before being cleaned by ethanol precipitation then quantified using the Qubit dsDNA HS Assay Kit (Q32854, Invitrogen/Life Technologies). An equimolar mix of all samples was prepared before carrying out a final clean up step using the Wizard SV Gel and PCR Clean-Up System (A9281, Promega). The equimolar mix was then eluted in 50 µl of Nuclease-Free Water, of which 25 µl was submitted for Illumina MiSeq sequencing (paired end 250 bp read length) at the Wellcome Sanger Institute in Cambridgeshire, UK.

#### Analysis of microbiota sequence data

Illumina MiSeq data was analyzed using mothur software (v 1.39.5)^61^, similar to as described by Dalby et al.^60^. Contigs were assembled using the forward and reverse reads, and only those which were between 280 and 470 bases were taken forward in the analysis pipeline. Sequences were aligned against the SILVA reference database and operational taxonomic units (OTUs) generated (97% similarity) using the default OptiClust option in mothur^62^. Representative sequences were obtained for each OTU and these were then able to be run through the BLAST database for species identification. Unlike Dalby et al. 2017^60^, no chimera removal software was used and instead a cut-off to remove all sequences with 10 reads or less applied. All samples were sub-sampled to 4237 reads to allow comparison across all samples at the same depth of coverage. The median Good’s coverage estimate at this sequence depth was 98.07% (range from 96.55 to 99.74%). Sequence data have been deposited in the European Nucleotide Archive under study accession number PRJEB14084. The final OTU table of the 500 most abundant OTU is shown as Supplementary Table 2, alongside results for each sample at the genus, family and phylum levels. Principal Coordinate Analysis (PCoA) plots, and dendrograms were created from the OTU-level data using the Bray Curtis dissimilarity calculator in mothur^62^.

### Quantitative reverse transcriptase-PCR

RNA was isolated using an Isolate II RNA micro kit (Bioline). cDNA was synthesized by means Superscript II reverse transcriptase (Invitrogen) for colon with oligo (dT) and random primers. A SYBR green-based real-time PCR technique was used to detect the expression of transcripts (SensiFAST master mix, GC-Biotech). Real-time PCR was performed using the Light cycler 480 (Roche) detection system. Data were analyzed using LinregPCR software^63^ and results were expressed as fold difference relative to the geometric mean expression of the reference genes ubiquitin and cyclophilin for murine colon. The following human primers sets were used:

β-actin F: AATGTGGCCGAGGACTTTGA, R: TGGCTTTTAGGATGGCAAGG, GAPDH F: GAGTCAACGGATTTGGTCGT, R: TTGATTTTGGAGGGATCTCG, mTNF F: TGGAACTGGCAGAAGAGGCACT, R: CCATAGAACTGATGAGAGGGAGGC, mIL-6 F: AGTTGCCTTCTTGGGACTGA, R: TCCACGATTTCCCAGAGAAC, mIL-1b F: GCCCATCCTCTGTGACTCAT, R: AGGCCACAGGTATTTTGTCG.

### Metabolome analysis

#### ^1^H Nuclear magnetic resonance (NMR) spectroscopy

Fecal samples were weighted and mixed with water (UPLC grade) in a ratio of 1 mg : 4 μl (fecal weight : water volume). The samples were mixed by pipetting, vortexing and centrifuged at 18,000*g* for 10 min at 4 °C. The fecal water supernatant was then separated from the pellet and stored at − 40 °C. Fecal water samples were thawed and centrifuged at 18,000*g* for 10 min at 4 °C. A total of 100 μl fecal water was mixed with 400 μl of deuterium oxide (D_2_O) and 100 μl of 0.2 M sodium phosphate buffer (pH = 7.4) containing 0.01% TSP (3-(trimethylsilyl)propionic-2,2,3,3-d_4_ acid sodium salt) and 3 mM NaN_3_. After vortexing, 580 μl was transferred to an NMR tube with an outer diameter of 5 mm pending ^1^H NMR spectral acquisition.

^1^H NMR spectra of fecal water samples were acquired using a Bruker 600 MHz spectrometer (Bruker, Rheinstetten, Germany) at the operating ^1^H frequency of 600.13 MHz at a temperature of 300 K. A standard NMR pulse sequence (recycle delay-90°-t_1_-90°-t_m_-90°-acquisition) was applied to acquire one-dimensional ^1^H NMR spectral data, where t_1_ was set to 3 μs and t_m_ (mixing time) was set to 10 ms. The water peak suppression was achieved using selective irradiation during a recycle delay of 2 s and t_m_. A 90° pulse was adjusted to ~ 10 μs. A total of 128 scans were collected into 64 k data points with a spectral width of 20 ppm. The standard parameters used for these spectral acquisitions have previously been reported^64,65^.

Metabolic data have been submitted to the MetaboLights database^66^ under accession number MTBLS2051.

### High-performance liquid chromatography (HPLC)

#### Amino acids

Amino acids were quantified in fecal water prepared by weighing the fecal sample and mixing it with four parts of sodium phosphate buffer [pH = 7.4], vortexing and centrifugation at 20,000*g* at 4 °C for 30 min. Fecal water (50 µl) was mixed with 24% sulfosalicylic acid and centrifuged at 20,000*g* at 4 °C to remove proteins. Amino acids were measured using a gradient reversed-phase HPLC system with precolumn derivatization with o-phtalaldehyde (Pierce) and 3-mercaptopropionic acid (Sigma), and fluorescence detection. Separation was done using 2 serial coupled BDS Hypersil C18 columns (150 × 4.8 mm, 3 µm particles, Thermo Scientific, flowrate 0.7 ml/min) and linear gradient of solvent A and B (from 10% B at start to 100% B at the end linearly). Solvent A was 12.5 mM sodium phosphate (pH 7.0) + 0.005% tetrahydrofuran and solvent B was 6 mM sodium phosphate (pH 7.0) + 0.07% tetrahydrofuran + 40% acetonitrile^67^. For normalization purposes we used norvaline as an internal standard.

#### Bile acids

Feces was freeze dried overnight. Feces were diluted 1:10 on a weight:volume basis with 50% Tert-Butanol solution, mixed, sonicated, and centrifuged at 3500* g*. Supernatant was freeze-dried overnight and re-suspended in 300 µl 25% Methanol. Bile acids were separated and quantified by reverse-phase HPLC, which was an adaptation to the method used by Kunne et al.^68^. 100 µl sample was applied to a Hypersil C18 HPLC column (internal diameter: 3 µm, column length: 15 cm; Thermo Scientific, Breda, The Netherlands) operated at 20 °C. The starting eluent consisted of 6.8 mM ammonium formiate (pH 3.9), followed by a linear gradient or isocratic elution with acetonitrile at the indicated concentration: 28% (1 min), 38% (13 min), 42% (19 min), 61% (20 min), 63% (25 min), 80% (28 min), 80% (31 min) and 0% (33 min). The flow rate was 0.8 mL per minute. Detection was performed using a Charged aerosol detector (ThermoFisher). Quantification of the different bile salt species was performed by using a calibration curve for all different bile salt species.

### Real time cell growth and adherence assay

The effect of metabolites on epithelial cell proliferation and adhesion was performed using a label-free real-time cell analysis platform (xCELLigence; Roche Applied Science, Indianapolis, IN) as previously described^69^. Each well of a 16 well E-plate received, 100 μl complete Dulbecco's Modified Eagle Medium (DMEM) culture medium supplemented with metabolites of interest, and background impedance was measured over 24 s. Caco-2 cells were cultured until confluent, trypsinized and suspended in DMEM supplemented at 20,000 cells/ml. Resuspended cells (100 μl) were added to the E-plate wells. After 30 min of incubation at room temperature, the plate was placed into the cell culture incubator (37 °C, 5% CO_2_). Control wells received DMEM medium only. Cell proliferation and adhesion was measured every 30 min for 72 h by determining the cell index using the xCELLigence system software version 1.2.1^70^.

### Monocyte immunological response assay

Primary monocytes were isolated from whole blood buffy coats in 2 steps: (1) Ficoll was added under the buffy coat layer and spun down (2000 RCF, acc. 3, decl. 0, 20 min) and the separated layer of peripheral blood mononuclear cells (PBMC) were aspirated an re-suspended, (2) PBMC were incubated (90 min, 37 °C, 5% CO_2_) after which culture plates were washed and remaining monocytes loosened with EDTA and re-suspended and plated at 500.000 cells/well in 6 well plates (1.5 ml/plate). Monocytes were co-cultured with metabolites of interest for 24 h, after which LPS (100 ng/ml) was added. The medium was subsequently collected after 4 h and Tumor Necrosis Factor-alpha (TNFα) and Interleukin-6 (IL-6) were measured using sandwich ELISA (R&D Systems, Minneapolis, Minn., USA).

### Dextran sulfate sodium (DSS) colitis mouse model

C57BL/6 N mice (Charles River Laboratories) were housed and maintained under specific pathogen free conditions in our animal facility at the AMC in Amsterdam. Mice were females between 8 and 12 weeks of age at the time of study. Eleven days prior to inducing intestinal inflammation with DSS, mice were given chow supplemented with milk protein (n = 10) or chow supplemented with amino acids (n = 10) (Mead Johnson Nutrition). Intestinal inflammation was induced using 1.5% (w/v) DSS (TdB Consultancy, Uppsala, Sweden) added to the drinking water for 7 days. Fresh DSS solutions were prepared daily. Body weights were recorded daily. At the end of the study endoscopy was done according to the scoring system described by Becker et al.^71^. Five features of endoscopic severity were scored: ‘mucosal thickening’, ‘vasculature’, ‘granularity of the mucosal surface’, ‘fibrin deposits’ and ‘stool appearance’. The total endoscopic disease severity score was calculated from the disease components, excluding the stool component score (as it was only clearly determinable in 158 of 201 of the videos), with a total score between 0 and 12. Subsequent to endoscopy mice were killed and organs collected. Wet weights of colons were recorded together with total colon length. Colon weight per cm was used as a disease parameter. Stools were scored as follows: (0) normal feces, (1) soft pellets, (2) thin feces, (3) watery diarrhea, (4) bloody diarrhea. Colons were divided in two parts longitudinally; one part was used for histology the other part for qPCR analysis.

#### Histology

Histology was performed as described previously^72^. Longitudinally divided colons were rolled, fixed in 4% formalin and embedded in paraffin for routine histology. An experienced pathologist evaluated formalin-fixed hematoxylin tissue sections microscopically, in a blinded fashion. Colons were evaluated, and graded from 0 to 4 as an indication of incidence and severity of inflammatory lesions based on the extent of the area involved, the number of follicle aggregates, edema, fibrosis, hyperplasia, erosion/ulceration, crypt loss and infiltration of granulocytes and mononuclear cells. The pathology score was calculated as the total score of the above.

### Statistical analysis

Microbiota and metabolomic data were compared between (1) CD patients at T0 and healthy controls, (2) between different time points during EEN in CD patients (T0 vs. T1 vs. T2 vs. T3), (3) between biochemical responders and non-responders, and (4) between responder or non-responders at follow-up (T3) and healthy controls.

The statistical analysis for the microbiota data was carried out as described by Dalby et al. 2017^60^, using LEfSe^73^ and Metastats^74^ (*p* values corrected with Benjamini–Hochberg to account for multiple comparisons)^75^ to assess changes in proportional abundance across OTUs, Genus, Family and Phylum Levels. Differences in overall community compositions were analyzed at the OTU level using the Analysis of Molecular Variance (AMOVA) function within the mothur software package^76^, based on Bray Curtis dissimilarity. The Shannon and inverse Simpson diversity indices, which are commonly used to characterizes species diversity in a community based on proportional abundance and evenness of the species present, were used to calculate the bacterial diversity within each sample using the mothur software package^76^. These were compared between cohorts using Mann–Whitney tests for CD versus healthy control comparisons, and using the Wilcoxon test for the longitudinal comparisons across the term of the EEN intervention. The subsets of samples that were included in each of the comparative analyses included in the Results section are shown in Supplementary Table 3.

Multivariate statistical analysis of ^1^H NMR spectral data were pre-processed (phasing, baseline correction and calibration) and imported into MATLAB (R2014a) using an in-house MATLAB script from Imperial College London. The shift ranges from − 0.02 to 0.02 ppm, 3.70 to 3.72 ppm and 4.78 to 4.84 ppm were removed to exclude TSP, polyethylene glycol (PEG) and water peaks, respectively. Due to the high intensity of PEG compared to other peaks in the spectrum, PEG was deemed to be a remnant of the bowel cleansing procedure present in some patients and therefore excluded from further analysis. NMR spectra were then aligned using recursive segment-wise peak alignment^77^, normalized using the probabilistic quotient normalization^78^ and log-transformed prior to Principal Component Analysis (PCA) and Orthogonal Partial Least Squares-Discriminant Analysis (OPLS-DA).

Unit variance scaling method and sevenfold cross validation were used in OPLS-DA models. The model parameters were presented as R^2^ (percentage of variation explained by the model) and Q^2^ (predictivity of the models) and *p* value generated from the permutation tests. Metabolites that are associated with CD, EEN treatment or EEN response were extracted from OPLS-DA models using statistical total correlation spectroscopy (STOCSY), the Human Metabolite Database and other literature documenting faecal water metabolites obtained from NMR^79–81^. Correlation coefficient values (r) were provided based on a selected signal from each metabolite. *p*- and q-values represent the significance of the metabolite changes and Benjamini–Hochberg correction-adjusted *p*-values, respectively.

Bile acids were categorized into primary and secondary bile acids. The hydrophobicity of the bile acid pool per fecal sample was calculated using the bile acid hydrophobicity index^82^. Differences in amino acids (individual and total concentration) and bile acids (primary and secondary concentration, and bile acid hydrophobicity index) were analyzed using *t* test (2 groups) and one-way ANOVA with Turkey post-hoc test (> 2 groups) for normally distributed or Mann–Whitney U (2 groups) and Kruskal–Wallis test with Dunn’s post-hoc test (> 2 groups) for non-normally distributed variables.

Cell indexes deriving from XCELLigence and cytokine concentrations deriving from the monocyte immunological response assay were analyzed identical to amino and bile acid data.

## Supplementary information

Legends for supplementary Figures 1–3.

Supplementary Tables 1, 4–12.

Supplementary Figure 1.

Supplementary Figure 2.

Supplementary Figure 3.

Supplementary Table 2.

Supplementary Table 3.
