# Supplementary material for: Exclusive enteral nutrition mediates gut microbial and metabolic changes that are associated with remission in children with Crohn’s disease
Source: Sci Rep. 2020 Nov 3;10:18879. doi: 10.1038/s41598-020-75306-z (PMC7609694; doi:10.1038/s41598-020-75306-z)
Supplement: Supplementary file 1 — Legends for supplementary Figures 1–3. [file 41598_2020_75306_MOESM1_ESM.docx]

Supplementary Figure 1

Individual impact of EEN on microbiota.

Bray Curtis dissimilarity dendrogram generated from the whole dataset OTU-level results showing the top 18 most prevalent bacterial families of Controls (Yellow), T0 (Green), T1 (Grey), T2 (Blue) and T3 (Purple).

The figure was generated using iTOL, version 5.6.3. (https://itol.embl.de/)

Supplementary Figure 2

ROC curves corresponding to OPLS-DA models of Figures 3A-C.

Panel A, ROC curve of OPLS-DA comparing the metabolome of HC and patients with CD at T0 as shown in Figure 3A

Panel B, ROC curve of OPLS-DA comparing the metabolome of patients with CD at T0 and at T2, as shown in Figure 3B

Panel C, ROC curve of OPLS-DA comparing responders and non-responders to EEN at T0, as shown in Figure 3C.

Supplementary Figure 3

Panel A, OPLS-DA comparing responders and non-responders to EEN at T3.

Panel B, Total plasma amino acid concentration versus fecal calprotectin in a cohort of patients with CD.

Panel C, Effect of TMA, TMAO and cadaverin on viability of PBMC.

Panels D-H, Composition of the fecal bile salt pool in controls and in patients with CD at different time points of EEN treatment.
