# Supplementary material for: Exclusive enteral nutrition mediates gut microbial and metabolic changes that are associated with remission in children with Crohn’s disease
Source: Sci Rep. 2020 Nov 3;10:18879. doi: 10.1038/s41598-020-75306-z (PMC7609694; doi:10.1038/s41598-020-75306-z)
Supplement: Supplementary file 2 — Supplementary Tables 1, 4–12. [file 41598_2020_75306_MOESM2_ESM.doc]

| **Supplementary table 1 │**  Composition of polymeric formulas used during exclusive enteral nutrition | | | | | | |
| --- | --- | --- | --- | --- | --- | --- |
|  | **Energy^a^**  (kcal/100ml)^a^ | **Saturated fat** (g)^b^ | **Monounsaturated fat** (g)^b^ | **Polyunsaturated fat** (g)^b^ | **Carbohydrates** (g)^b^ | **Protein**  (g)^b^ |
| Ensure Plus | 150 | 0.30 | 1.57 | 1.23 | 13.5 | 4.17 |
| Paediasure | 101 | 0.87 | 1.57 | 0.71 | 7.7 | 1.87 |
| Resource energy^c^ | 150 | 0.47 | 1.27 | 1.53 | 14.0 | 3.73 |
| Nutridrink | 150 | 0.40 | 2.33 | 1.13 | 12.3 | 3.93 |
| Fresubin Energy Drink | 150 | 0.27 | 2.53 | 1.07 | 12.5 | 3.73 |
| Paediasure Plus | 151 | 1.33 | 2.33 | 1.04 | 11.2 | 2.80 |
| Fresubin 2.0 kcal Drink | 200 | 0.39 | 3.85 | 0.95 | 15.0 | 6.67 |
| Resource 2.0 | 200 | 0.47 | 3.80 | 1.53 | 14.3 | 6.00 |
| Nutridrink compact | 240 | 0.60 | 3.80 | 1.80 | 19.8 | 6.40 |
| ^a^ energy per 100ml  ^b^ relative to the caloric value per 100ml  ^c^ additionally contains 0,33g of fibers  g: grams, kcal: kilocalories, ml: milliliter | | | | | | |

**Supplementary table 4:** Table showing OTUs, and their proportional abundances, that were significantly different between fecal samples from control and CD patient at baseline (T0).

| **OTU** | **NCBI Blast-based identification (% similarity)** | **Mean proportional abundance (%) in HC** | **Mean proportional abundance (%) in CD at T0** | **Metastats-based p-value (Benjamini-Hochberg corrected)** | **LEfSe p-value** |
| --- | --- | --- | --- | --- | --- |
| Otu0006 | *Dorea longicatena* (100%) | 0.99 | 2.45 | 0.023 | 0.011 |
| Otu0007 | *Blautia wexlerae/luti* (98%) | 1.13 | 3.81 | 0.014 | 0.004 |
| Otu0008 | *Escherichia coli* (100%) | 0.28 | 4.02 | 0.014 | <0.001 |
| Otu0009 | *Blautia luti* (99%) | 1.17 | 3.12 | 0.014 | 0.005 |
| Otu0013 | *Ruminococcus gnavus* (99%) | 0.01 | 2.23 | 0.023 | 0.012 |
| Otu0004 | *Bifidobacterium longum* (99%) | 5.98 | 2.07 | 0.014 | <0.001 |
| Otu0024 | *Ruminococcus bromii* (99%) | 2.46 | 0.19 | 0.035 | <0.001 |
| Otu0051 | *Eubacterium rectale* (100%) | 1.98 | 0.35 | 0.014 | <0.001 |

**Supplementary Table 5:** Tables showing OTUs, and their proportional abundances, that were identified by LEfSe as being associated with either healthy controls (HC), Responders (R) or Non-responders (NR) at both T0 (Table a) and T3 (Table b). LDA = Linear discriminant analysis, which is a LEfSe generated measure of effect size. LDA scores higher than 2 are typically considered to be noteworthy. (N. Segata, J. Izard, L. Waldron, D. Gevers, L. Miropolsky, W. S. Garrett & Curtis Huttenhower. Genome Biology volume 12, (2011))

Table a (T0)

| **OTU** | **NCBI Blast-based Identification (% similarity to closest related cultured species)** | **Mean proportional abundance (%) in HC** | **Mean proportional abundance (%) in R at T0** | **Mean proportional abundance (%) in NR at T0** | **Associated with HC, R or NR?** | **LDA score** | **LEfSe p-value** |
| --- | --- | --- | --- | --- | --- | --- | --- |
| Otu0002 | *Holdemanella biformis* (99%) | 2.08 | 0.01 | 9.29 | NR | 4.689 | 0.033 |
| Otu0003 | “Guyana massiliensis” (100%) | 1.41 | 2.38 | 6.35 | NR | 4.352 | 0.042 |
| Otu0004 | *Bifidobacterium longum* (99%) | 5.98 | 0.77 | 2.68 | HC | 4.398 | <0.001 |
| Otu0006 | *Dorea longicatena* (100%) | 0.99 | 0.85 | 3.08 | NR | 4.056 | <0.001 |
| Otu0008 | *Escherichia coli* (100%) | 0.28 | 2.12 | 3.42 | NR | 4.197 | <0.001 |
| Otu0011 | No hits >95% to cultured species | 2.98 | 5.54 | 0.62 | R | 4.502 | 0.046 |
| Otu0012 | *Romboutsia timonensis* (99%) | 5.58 | 2.41 | 0.80 | HC | 4.419 | 0.011 |
| Otu0013 | *Ruminococcus gnavus* (99%) | 0.01 | 8.40 | 0.26 | R | 4.489 | 0.007 |
| Otu0020 | *Blautia obeum* (99%) | 0.52 | 0.25 | 1.97 | NR | 3.863 | 0.002 |
| Otu0024 | *Ruminococcus bromii* (99%) | 2.46 | 0.46 | 0.11 | HC | 4.055 | 0.013 |
| Otu0026 | *Bifidobacterium pseudocatenulatum* (100%) | 3.69 | 0.07 | 0.92 | HC | 4.258 | 0.043 |
| Otu0027 | *Faecalibacterium prausnitzii* (97%) | 2.07 | 0.52 | 1.21 | HC | 3.782 | 0.044 |
| Otu0030 | *Anaerobutyricum hallii* (99%) | 0.13 | 0.33 | 0.98 | NR | 3.591 | 0.021 |
| Otu0035 | *Bifidobacterium bifidum* (100%) | 1.15 | 2.56 | 0.11 | R | 4.015 | 0.033 |
| Otu0043 | No hits >95% to cultured species | 0.25 | 0.06 | 0.69 | NR | 3.494 | 0.010 |
| Otu0045 | *Ruminococcus torques* (100%) | 0.05 | 0.21 | 0.23 | NR | 2.997 | 0.036 |
| Otu0047 | *Blautia obeum* (97%) | 0.23 | 0.01 | 0.97 | NR | 3.601 | 0.001 |
| Otu0051 | *Eubacterium rectale* (100%) | 1.98 | 0.31 | 0.43 | HC | 3.882 | 0.003 |
| Otu0052 | *Intestinibacter bartlettii* (97%) | 0.67 | 0.31 | 0.13 | HC | 3.482 | 0.025 |
| Otu0055 | Butyrate-producing bacterium A2-231 (100%) | 0.20 | 0.31 | 0.63 | NR | 3.332 | 0.007 |
| Otu0059 | *Peptoniphilus tyrrelliae* (99%) | 0.00 | 4.49 | 0.07 | R | 4.253 | 0.009 |
| Otu0062 | *Clostridium saccharoperbutylacetonicum* (96%) | 1.77 | 0.45 | 0.04 | HC | 3.913 | 0.001 |
| Otu0064 | *Enterococcus faecium* (100%) | 0.01 | 0.29 | 0.16 | R | 3.143 | 0.035 |
| Otu0070 | *Dorea longicatena* (98%) | 0.11 | 0.00 | 0.37 | NR | 3.252 | <0.001 |
| Otu0082 | *Gemella morbillorum* (100%) | 0.00 | 1.97 | 0.06 | R | 3.891 | 0.032 |
| Otu0083 | *Ruminococcus* sp. (99%) | 0.08 | 0.06 | 0.32 | NR | 3.104 | 0.002 |
| Otu0084 | *Coprococcus comes* (97%) | 0.11 | 0.24 | 0.53 | NR | 3.341 | 0.005 |
| Otu0087 | *Clostridium saudiense* (99%) | 0.66 | 0.59 | 0.05 | HC | 3.512 | 0.003 |
| Otu0089 | *Romboutsia lituseburensis* (99%) | 0.71 | 0.19 | 0.11 | HC | 3.549 | 0.005 |
| Otu0091 | *Finegoldia magna* (100%) | 0.00 | 0.07 | 0.01 | R | 2.496 | 0.024 |
| Otu0100 | *Ruminococcus* sp. (96%) | 0.00 | 0.02 | 0.09 | NR | 2.688 | 0.037 |
| Otu0102 | *Granulicatella adiacens* (99%) | 0.01 | 1.41 | 0.06 | R | 3.743 | 0.018 |
| Otu0114 | No hits >95% to cultured species | 0.05 | 0.04 | 0.26 | NR | 3.077 | 0.039 |
| Otu0128 | *Bacteroides uniformis* (100%) | 0.08 | 0.00 | 0.10 | NR | 2.776 | 0.043 |
| Otu0130 | *Subdoligranulum variabile* (98%) | 0.29 | 0.00 | 0.11 | HC | 3.123 | 0.019 |

Table b (T3):

| **OTU** | **NCBI Blast-based Identification (% similarity to closest related cultured species)** | **Mean proportional abundance (%) in HC** | **Mean proportional abundance (%) in R at T3** | **Mean proportional abundance (%) in NR at T3** | **Associated with HC, R or NR?** | **LDA score** | **LEfSe p-value** |
| --- | --- | --- | --- | --- | --- | --- | --- |
| Otu0006 | *Dorea longicatena* (100%) | 0.99 | 1.48 | 2.65 | NR | 3.950 | 0.011 |
| Otu0007 | *Blautia wexlerae/luti* (98%) | 1.13 | 2.92 | 3.40 | NR | 3.951 | 0.003 |
| Otu0008 | *Escherichia coli* (100%) | 0.28 | 0.28 | 2.34 | NR | 3.938 | 0.004 |
| Otu0009 | *Blautia luti* (99%) | 1.17 | 2.53 | 3.20 | NR | 3.923 | 0.008 |
| Otu0011 | No hits >95% to cultured species | 2.98 | 8.58 | 0.52 | R | 4.660 | 0.006 |
| Otu0017 | *Bifidobacterium adolescentis* (100%) | 4.63 | 6.73 | 0.23 | R | 4.588 | 0.032 |
| Otu0020 | *Blautia obeum* (99%) | 0.52 | 0.36 | 2.61 | NR | 4.046 | 0.026 |
| Otu0022 | *Bifidobacterium adolescentis/faecale* (100%) | 4.73 | 0.19 | 0.51 | HC | 4.375 | 0.042 |
| Otu0024 | *Ruminococcus bromii* (99%) | 2.46 | 13.73 | 0.36 | R | 4.752 | 0.009 |
| Otu0027 | *Faecalibacterium prausnitzii* (97%) | 2.07 | 2.17 | 0.27 | R | 4.004 | 0.034 |
| Otu0045 | *Ruminococcus torques* (100%) | 0.05 | 0.00 | 0.11 | NR | 2.665 | 0.045 |
| Otu0047 | *Blautia obeum* (97%) | 0.23 | 0.04 | 0.71 | NR | 3.484 | 0.009 |
| Otu0051 | *Eubacterium rectale* (100%) | 1.98 | 1.44 | 0.11 | HC | 3.963 | 0.003 |
| Otu0058 | *Clostridiaceae* bacterium DJF_LS40 (99%) | 0.52 | 1.59 | 0.16 | R | 3.897 | 0.027 |
| Otu0062 | *Clostridium saccharoperbutylacetonicum* (96%) | 1.77 | 0.26 | 0.27 | HC | 3.861 | 0.043 |
| Otu0083 | *Ruminococcus* sp. (99%) | 0.08 | 0.11 | 0.27 | NR | 3.023 | 0.013 |
| Otu0084 | *Coprococcus comes* (97%) | 0.11 | 0.02 | 0.37 | NR | 3.242 | 0.003 |
| Otu0087 | *Clostridium saudiense* (99%) | 0.66 | 0.06 | 0.07 | HC | 3.412 | 0.010 |
| Otu0100 | *Ruminococcus* sp. (96%) | 0.00 | 0.07 | 0.05 | R | 2.542 | 0.035 |

| **Supplementary table 6 │**  Individual fecal metabolites elevated in CD patients at T0, differences between controls and responders or non-responders at T3. | | | | | | |
| --- | --- | --- | --- | --- | --- | --- |
|  | **T0: Patients (n=43) versus Controls (n=31)** | | **T3: Responders (n=7) versus Controls(n=31)** | | **T3: Non-responders (n=10) versus Controls (n=31)** | |
|  | **r^a^** | **p^b^** | **r^a^** | **p^b^** | **r^a^** | **p^b^** |
| Alanine | 0.60 | <0.001 | 0.38 | 0.234 | 0.52 | 0.001 |
| Cadaverine | 0.64 | <0.001 | 0.51 | 0.069 | 0.76 | <0.001 |
| Isoleucine | 0.57 | <0.001 | 0.29 | 0.413 | 0.43 | 0.007 |
| Lactate | 0.58 | <0.001 | 0.51 | 0.072 | 0.50 | 0.002 |
| Leucine | 0.62 | <0.001 | 0.34 | 0.304 | 0.53 | <0.001 |
| Phenylalanine | 0.66 | <0.001 | 0.41 | 0.190 | 0.63 | <0.001 |
| Propionate | 0.56 | <0.001 | 0.40 | 0.203 | 0.69 | <0.001 |
| Putrescine | 0.53 | <0.001 | 0.67 | 0.008 | 0.64 | <0.001 |
| Trimethylamine | 0.63 | <0.001 | 0.25 | 0.497 | 0.65 | <0.001 |
| Tryptophan | 0.64 | <0.001 | 0.59 | 0.027 | 0.59 | <0.001 |
| Tyrosine | 0.60 | <0.001 | 0.36 | 0.268 | 0.61 | <0.001 |
| Valine | 0.63 | <0.001 | 0.38 | 0.234 | 0.50 | 0.002 |
| Fecal metabolites higher in patients at T0 compared to controls were identified by ^1^H NMR. In responders only at T3, many of these metabolites were not different from controls.  ^a^ Pearson correlation (r) +/- high/low in CD ^b^ Benjamini-Hochberg corrected p value | | | | | | |

| **Supplementary Table 7 │**  Fecal amino acid concentration in controls and patients at T0-T3. | | | | | | | |
| --- | --- | --- | --- | --- | --- | --- | --- |
|  | **Healthy controls**  **(n=31)** | **Baseline (T0)**  **(n=43)** | **During EEN (T1)**  **(n=37)** | **End EEN (T2)**  **(n=22)** | **Follow-up (T3)**  **(n=20)** | **P value^a^** | **Post-hoc test^b^** |
| Aspartic acid  *(µM, median (IQR))* | 372 (276−516) | 628 (339−947) | 475 (335−787) | 625 (297−800) | 461 (307−760) | 0.0277 | HC vs T0: Yes  T0 vs T1: No  T0 vs T2: No  T0 vs T3: No  T1 vs T2: No  T1 vs T3: No  T2 vs T3: No |
| Glutamic acid  *(µM, median (IQR))* | 954 (740−1260) | 1487 (889−1967) | 1000 (732−1344) | 1044 (550−1449) | 1179 (889−1839) | 0.0519 | NA |
| Asparagine  *(µM, median (IQR))* | 1 (0−5) | 27 (5−74) | 16 (6−66) | 17 (7−54) | 22 (6−69) | <0.0001 | HC vs T0: Yes  T0 vs T1: No  T0 vs T2: No  T0 vs T3: No  T1 vs T2: No  T1 vs T3: No  T2 vs T3: No |
| Serine  *(µM, median (IQR))* | 184 (129−258) | 395 (274−592) | 357 (228−482) | 438 (299−559) | 405 (194−543) | <0.0001 | HC vs T0: Yes  T0 vs T1: No  T0 vs T2: No  T0 vs T3: No  T1 vs T2: No  T1 vs T3: No  T2 vs T3: No |
| Glutamine  *(µM, median (IQR))* | 61 (49−94) | 131 (77−206) | 113 (59−160) | 109 (71−173) | 124 (71−187) | 0.0001 | HC vs T0: Yes  T0 vs T1: No  T0 vs T2: No  T0 vs T3: No  T1 vs T2: No  T1 vs T3: No  T2 vs T3: No |
| Histidine  *(µM, median (IQR))* | 33 (15−53) | 73 (50−119) | 50 (34−69) | 66 (38−95) | 50 (35−89) | <0.0001 | HC vs T0: Yes  T0 vs T1: No  T0 vs T2: No  T0 vs T3: No  T1 vs T2: No  T1 vs T3: No  T2 vs T3: No |
| Glycine  *(µM, median (IQR))* | 301 (210−481) | 711 (423−1149) | 442 (283−756) | 592 (402−968) | 676 (347−1086) | <0.0001 | HC vs T0: Yes  T0 vs T1: No  T0 vs T2: No  T0 vs T3: No  T1 vs T2: No  T1 vs T3: No  T2 vs T3: No |
| Threonine  *(µM, median (IQR))* | 159 (129−213) | 344 (200−507) | 279 (185−423) | 352 (196−500) | 301 (174−577) | <0.0001 | HC vs T0: Yes  T0 vs T1: No  T0 vs T2: No  T0 vs T3: No  T1 vs T2: No  T1 vs T3: No  T2 vs T3: No |
| Citruline  *(µM, median (IQR))* | 151 (96−231) | 365 (193−577) | 241 (163−398) | 297 (215−410) | 342 (222−522) | <0.0001 | HC vs T0: Yes  T0 vs T1: No  T0 vs T2: No  T0 vs T3: No  T1 vs T2: No  T1 vs T3: No  T2 vs T3: No |
| Arginine  *(µM, median (IQR))* | 59 (28−119) | 59 (32−141) | 70 (35−107) | 39 (30−109) | 26 (11−160) | 0.6317 | NA |
| Alanine  *(µM, median (IQR))* | 742 (504−948) | 1505 (934−2138) | 1092 (776−1478) | 1348 (1011−1756) | 1318 (664−1766) | <0.0001 | HC vs T0: Yes  T0 vs T1: No  T0 vs T2: No  T0 vs T3: No  T1 vs T2: No  T1 vs T3: No  T2 vs T3: No |
| Taurine  *(µM, median (IQR))* | 34 (21−54) | 56 (26−239) | 30 (18−80) | 38 (20−169) | 57 (22−220) | 0.1464 | NA |
| Tyrosine  *(µM, median (IQR))* | 168 (116−248) | 370 (228−512) | 325 (178−417) | 351 (261−436) | 350 (173−496) | <0.0001 | HC vs T0: Yes  T0 vs T1: No  T0 vs T2: No  T0 vs T3: No  T1 vs T2: No  T1 vs T3: No  T2 vs T3: No |
| Valine  *(µM, median (IQR))* | 296 (227−493) | 834 (471−1112) | 599 (401−767) | 709 (422−827) | 740 (401−972) | 0,0004 | HC vs T0: Yes  T0 vs T1: No  T0 vs T2: No  T0 vs T3: No  T1 vs T2: No  T1 vs T3: No  T2 vs T3: No |
| Methionine  *(µM, median (IQR))* | 116 (76−158) | 227 (127−297) | 152 (126−254) | 198 (118−283) | 203 (94−281) | <0.0001 | HC vs T0: Yes  T0 vs T1: No  T0 vs T2: No  T0 vs T3: No  T1 vs T2: No  T1 vs T3: No  T2 vs T3: No |
| Isoleucine  *(µM, median (IQR))* | 246 (187−412) | 623 (394−886) | 440 (327−608) | 526 (273−678) | 555 (280−718) | <0.0001 | HC vs T0: Yes  T0 vs T1: No  T0 vs T2: No  T0 vs T3: No  T1 vs T2: No  T1 vs T3: No  T2 vs T3: No |
| Tryptophan  *(µM, median (IQR))* | 27 (20−32) | 72 (45−106) | 55 (35−87) | 56 (43−76) | 64 (37−120) | <0.0001 | HC vs T0: Yes  T0 vs T1: No  T0 vs T2: No  T0 vs T3: No  T1 vs T2: No  T1 vs T3: No  T2 vs T3: No |
| Phenylalanine  *(µM, median (IQR))* | 180 (126−272) | 454 (267−620) | 352 (204−496) | 425 (308−487) | 397 (223−657) | <0.0001 | HC vs T0: Yes  T0 vs T1: No  T0 vs T2: No  T0 vs T3: No  T1 vs T2: No  T1 vs T3: No  T2 vs T3: No |
| Leucine  *(µM, median (IQR))* | 300 (212−471) | 759 (455−1009) | 602 (350−771) | 701 (453−779) | 692 (342−989) | <0.0001 | HC vs T0: Yes  T0 vs T1: No  T0 vs T2: No  T0 vs T3: No  T1 vs T2: No  T1 vs T3: No  T2 vs T3: No |
| Lysine  *(µM, median (IQR))* | 480 (362−694) | 908 (553−1166) | 672 (445−1004) | 839 (517−1022) | 791 (503−1199) | 0,0002 | HC vs T0: Yes  T0 vs T1: No  T0 vs T2: No  T0 vs T3: No  T1 vs T2: No  T1 vs T3: No  T2 vs T3: No |
| Total amino acids  *(µM, median (IQR))* | 4953 (3937−7146) | 10745 (7241−13668) | 7586 (5096−9990) | 9479 (6373−11508) | 9506 (5699−12475) | <0.0001 | HC vs T0: Yes  T0 vs T1: No  T0 vs T2: No  T0 vs T3: No  T1 vs T2: No  T1 vs T3: No  T2 vs T3: No |
| Fecal Amino acids are expressed in µM and measured by HPLC. Concentrations between controls and patients were different at all time points except glutamic acid, arginine and taurine.  **^a^** Kruskall-Wallis test  **^b^** Dunn’s multiple comparison post-hoc test. | | | | | | | |

| **Supplementary Table 8 │**  Fecal amino acid concentrations in responders versus non-responders before and during EEN | | | | | | | | |
| --- | --- | --- | --- | --- | --- | --- | --- | --- |
|  | **Baseline (T0)** | | **During EEN (T1)** | | **End EEN (T2)** | | **Follow-up (T3)** | |
|  | **Responders**  **(n=10) vs**  **Non-responders (n=12)** | **p** | **Responders**  **(n=10) vs**  **Non-responders (n==11)** | **p** | **Responders**  **(n=7) vs**  **Non-responders (n=11)** | **p** | **Responders**  **(n=7) vs**  **Non-responders (n=10)** | **p** |
| Aspartic acid  *(µM, median (IQR))* | 244 (653-1141) vs 520 (874-1195) | 0.381 | 308 (526-674) vs 289 (414-735) | 0.918 | 649 (244-885) vs  656 (468-795) | 0,556 | 455 (214-763) vs  567 (364-1012) | 0.495 |
| Glutamic acid  *(µM, median (IQR))* | 383 (1710-2006) vs 1446 (1924-2241) | 0.228 | 734 (870-1075) vs 538 (1176-1405) | 0.197 | 1274 (436-1424) vs 1045 (957-1524) | 0,684 | 1421 (985-1904) vs 1071 (813-1397) | 0.435 |
| Asparagine  *(µM, median (IQR))* | 5 (23-150) vs 4 (40-119) | 0.722 | 6 (9-32) vs 5 (48-90) | 0.173 | 51 (15-55) vs  13 (4-104) | 0,189 | 29 (6-114) vs  19 (1-60) | 0.495 |
| Serine  *(µM, median (IQR))* | 213 (574-889) vs 354 (526-827) | 0.872 | 181 (407-458) vs 264 (375-456) | 0.973 | 405 (183-449) vs  521 (364-709) | 0,033* | 417 (147-501) vs  413 (249-780) | 0.558 |
| Glutamine  *(µM, median (IQR))* | 63 (152-279) vs 96 (190-288) | 0.582 | 56 (108-166) vs 85 (114-145) | 1.000 | 105 (73-161) vs  153 (67-251) | 0,497 | 121 (71-141) vs  143 (87-243) | 0.380 |
| Histidine  *(µM, median (IQR))* | 21 (51-202) vs 66 (79-178) | 0.123 | 13 (48-58) vs 31 (48-67) | 0.605 | 63 (38-87) vs  83 (39-107) | 0,390 | 69 (38-223) vs  47 (35-81) | 0.380 |
| Glycine  *(µM, median (IQR))* | 156 (703-961) vs 509 (1039-1305) | 0.180 | 219 (442-590) vs 267 (426-530) | 0.973 | 430 (149-606) vs  881 (498-1100) | 0,026* | 1001 (476-1296) vs 660 (313-957) | 0.283 |
| Threonine  *(µM, median (IQR))* | 170 (416-714) vs 296 (459-726) | 0.628 | 173 (216-380) vs 236 (306-422) | 0.557 | 318 (130-413) vs  456 (312-509) | 0,063 | 335 (199-577) vs  355 (200-628) | 0.770 |
| Citruline  *(µM, median (IQR))* | 117 (292-554) vs 311 (506-614) | 0.059 | 169 (221-309) vs 174 (286-403) | 0.468 | 215 (200-416) vs  318 (283-461) | 0,135 | 355 (97-541) vs  296 (224-534) | 0.696 |
| Arginine  *(µM, median (IQR))* | 51 (91-367) vs 50 (80-141) | 0.582 | 27 (53-85) vs 44 (80-151) | 0.173 | 37 (26-170) vs  76 (38-115) | 0,684 | 14 (7-69) vs  94 (23-185) | 0.097 |
| Alanine  *(µM, median (IQR))* | 569 (1606-2023) vs 1188 (1978-2563) | 0.314 | 803 (959-1290) vs 794 (920-1383) | 1.000 | 1078 (479-1315) vs 1705 (1315-1975) | 0,010* | 1537 (607-1781) vs 1296 (873-1932) | 1.000 |
| Taurine  *(µM, median (IQR))* | 31 (171-333) vs 29 (68-244) | 0.722 | 14 (20-75) vs 21 (71-345) | 0.085 | 34 (18-204) vs  38 (26-120) | 0,751 | 58 (42-250) vs  32 (21-145) | 0.329 |
| Tyrosine  *(µM, median (IQR))* | 227 (344-623) vs 312 (445-597) | 0.628 | 163 (289-346) vs 205 (341-426) | 0.349 | 391 (207-423) vs  430 (293-466) | 0,135 | 410 (168-636) vs  338 (237-468) | 0.626 |
| Valine  *(µM, median (IQR))* | 395 (827-1142) vs 546 (1014-1310) | 0.381 | 380 (599-739) vs 405 (567-764) | 0.973 | 668 (201-758) vs  783 (590-904) | 0,063 | 970 (406-1087) vs 704 (470-941) | 0.495 |
| Methionine  *(µM, median (IQR))* | 103 (242-326) vs 148 (294-388) | 0.254 | 127 (152-222) vs 117 (173-259) | 0.918 | 129 (109-268) vs  249 (178-301) | 0,052 | 240 (78-284) vs  203 (108-289) | 0.922 |
| Isoleucine  *(µM, median (IQR))* | 235 (701-887) vs 473 (808-929) | 0.228 | 329 (414-563) vs 323 (415-597) | 0.863 | 481 (165-570) vs  606 (492-738) | 0,052 | 650 (186-795) vs  555 (392-785) | 0.845 |
| Tryptophan  *(µM, median (IQR))* | 42 (80-136) vs 68 (83-119) | 0.821 | 34 (46-78) vs 37 (58-72) | 0.863 | 58 (51-71) vs  74 (40-98) | 0,556 | 117 (49-171) vs  39 (31-106) | 0.079 |
| Phenylalanine  *(µM, median (IQR))* | 230 (515-697) vs 348 (553-811) | 0.628 | 182 (322-452) vs 240 (349-475) | 0.809 | 435 (233-462) vs  481 (353-543) | 0,135 | 661 (315-888) vs  397 (245-601) | 0.283 |
| Leucine  *(µM, median (IQR))* | 401 (761-1148) vs 548 (946-1228) | 0.456 | 348 (516-681) vs 395 (577-728) | 0.809 | 655 (242-744) vs  744 (636-856) | 0,094 | 832 (356-1177) vs 665 (417-927) | 0.495 |
| Lysine  *(µM, median (IQR))* | 423 (854-1510) vs 764 (1070-1391) | 0.497 | 594 (672-802) vs 418 (730-1120) | 0.654 | 662 (404-929) vs  901 (596-1192) | 0,135 | 654 (321-1197) vs 791 (568-1045) | 0.696 |
| Total amino acids  *(µM, median (IQR))* | 4922 (11082-15307) vs 9239 (12876-16481) | 0.381 | 4986 (6774-8896) vs 5799 (7741-10031) | 0.605 | 8503 (3881-10326) vs 11107 (8446-12036) | 0,052 | 10490 (4955-12752) vs 8955 (5871-12852) | 0.626 |
| Fecal Amino acids are expressed in µM and measured by HPLC. At T0, concentrations of Histidine, Citrulline and Isoleucine were different between responders and non-responders. At T3 Serine, Glycine and Alanine were different between responders and non-responders.  **^a^** Kruskall-Wallis test  **^b^** Dunn’s multiple comparison post-hoc test.  *IQR: inter quartile range* | | | | | | | | |

| **Supplementary Table 9 │**  Fecal amino acid concentrations in controls versus responders and non-responders at T3 | | | | | |
| --- | --- | --- | --- | --- | --- |
|  | **Healthy controls (n=32)** | **Responders (T3) (n=10)** | **Non-responders (T3) (n=7)** | **p^a^** | **Post-hoc test^b^** |
| Aspartic acid  *(µM, median (IQR))* | 372 (276−516) | 455 (214-763) | 567 (364-1012) | 0.3783 | HC vs R: no  HC vs NR: no |
| Glutamic acid  *(µM, median (IQR))* | 954 (740−1260) | 1421 (985-1904) | 1071 (813-1397) | 0.3721 | HC vs R: no  HC vs NR: no |
| Asparagine  *(µM, median (IQR))* | 1 (0−5) | 29 (6-114) | 19 (1-60) | 0.0015 | HC vs R: yes  HC vs NR: yes |
| Serine  *(µM, median (IQR))* | 184 (129−258) | 417 (147-501) | 413 (249-780) | 0.0115 | HC vs R: no  HC vs NR: yes |
| Glutamine  *(µM, median (IQR))* | 61 (49−94) | 121 (71-141) | 143 (87-243) | 0.2282 | HC vs R: no  HC vs NR: no |
| Histidine  *(µM, median (IQR))* | 33 (15−53) | 69 (38-223) | 47 (35-81) | 0.0159 | HC vs R: no  HC vs NR: yes |
| Glycine  *(µM, median (IQR))* | 301 (210−481) | 1001 (476-1296) | 660 (313-957) | 0.2501 | HC vs R: no  HC vs NR: no |
| Threonine  *(µM, median (IQR))* | 159 (129−213) | 335 (199-577) | 355 (200-628) | 0.0694 | HC vs R: no  HC vs NR: no |
| Citruline  *(µM, median (IQR))* | 151 (96−231) | 355 (97-541) | 296 (224-534) | 0.0922 | HC vs R: no  HC vs NR: no |
| Arginine  *(µM, median (IQR))* | 59 (28−119) | 14 (7-69) | 94 (23-185) | 0.9817 | HC vs R: no  HC vs NR: no |
| Alanine  *(µM, median (IQR))* | 742 (504−948) | 1537 (607-1781) | 1296 (873-1932) | 0.0667 | HC vs R: no  HC vs NR: no |
| Taurine  *(µM, median (IQR))* | 34 (21−54) | 58 (42-250) | 32 (21-145) | 0.007 | HC vs R: yes  HC vs NR: no |
| Tyrosine  *(µM, median (IQR))* | 168 (116−248) | 410 (168-636) | 338 (237-468) | 0.004 | HC vs R: no  HC vs NR: yes |
| Valine  *(µM, median (IQR))* | 296 (227−493) | 970 (406-1087) | 704 (470-941) | 0.0844 | HC vs R: no  HC vs NR: no |
| Methionine  *(µM, median (IQR))* | 116 (76−158) | 240 (78-284) | 203 (108-289) | 0.0807 | HC vs R: no  HC vs NR: no |
| Isoleucine  *(µM, median (IQR))* | 246 (187−412) | 650 (186-795) | 555 (392-785) | 0.2543 | HC vs R: no  HC vs NR: no |
| Tryptophan  *(µM, median (IQR))* | 27 (20−32) | 117 (49-171) | 39 (31-106) | < 0.0001 | HC vs R: yes  HC vs NR: yes |
| Phenylalanine  *(µM, median (IQR))* | 180 (126−272) | 661 (315-888) | 397 (245-601) | 0.0007 | HC vs R: no  HC vs NR: yes |
| Leucine  *(µM, median (IQR))* | 300 (212−471) | 832 (356-1177) | 665 (417-927) | 0.0211 | HC vs R: no  HC vs NR: yes |
| Lysine  *(µM, median (IQR))* | 480 (362−694) | 654 (321-1197) | 791 (568-1045) | 0.1333 | HC vs R: no  HC vs NR: no |
| Total amino acids  *(µM, median (IQR))* | 4953 (3937−7146) | 10490 (4955-12752) | 8955 (5871-12852) | 0.0892 | HC vs R: no  HC vs NR: no |
| Fecal Amino acids are expressed in µM and measured by HPLC. At T3 concentrations of Asparagine and Tryptophan remained different between both responders and non-responders and controls whereas most other amino acids were not different between responders and non-responders and controls.  ***^a^*** *Kruskall-Wallis test*  ***^b^*** *Dunn’s multiple comparison post-hoc test.*  *CD: Crohn’s disease, EEN: exclusive enteral nutrition, IQR: inter quartile range, NR: non-responders, R=responders* | | | | | |

| **Supplementary Table 10 │**  Fecal bile acid composition in controls and patients at T0-T3. | | | | | | | |
| --- | --- | --- | --- | --- | --- | --- | --- |
|  | **Healthy controls**  **(n=31)** | **Baseline (T0)**  **(n=43)** | **During EEN (T1)**  **(n=37)** | **End EEN (T2)**  **(n=22)** | **Follow-up (T3)**  **(n=20)** | **p^a^** | **Post-hoc test^b^** |
| Total bile acid concentration *(mM, median (IQR))* | 0.84 (0.42−1.42) | 0.95 (0.43−1.80) | 1.08 (0.58−1.95) | 0.92 (0.53−2.37) | 1.88 (0.71−2.92) | 0.0884 | HC vs T0: No  T0 vs T1: No  T0 vs T2: No  T0 vs T3: No  T1 vs T2: No  T1 vs T3: No  T2 vs T3: No |
| Bile acid hydrophobicity index *(median (IQR))* | 0.79 (0.73−0.81) | 0.73 (0.35−0.73) | 0.72 (0.61−0.74) | 0.74 (0.67−0.78) | 0.68 (0.45−0.77) | <0.0001 | HC vs T0: Yes  T0 vs T1: No  T0 vs T2: No  T0 vs T3: No  T1 vs T2: No  T1 vs T3: No  T2 vs T3: No |
| % secondary bile acids *(median (IQR))* | 93.7% (85.3−96.3) | 86.1% (46.9−94.7) | 91.9% (79.1−96.5) | 94.1% (88.3−98.1) | 88.6% (36.4−94.3) | 0.0498 | HC vs T0: No  T0 vs T1: No  T0 vs T2: No  T0 vs T3: No  T1 vs T2: No  T1 vs T3: No  T2 vs T3: No |
| ***^a^*** *Kruskall-Wallis test*  ***^b^*** *Dunn’s multiple comparison post-hoc test.*  *CD: Crohn’s disease, EEN: exclusive enteral nutrition, IQR: inter quartile range* | | | | | | | |

| **Supplementary Table 11 │**  Fecal bile acid composition in responders versus non-responders before and during EEN | | | | | | | | |
| --- | --- | --- | --- | --- | --- | --- | --- | --- |
|  | **Baseline (T0)** | | **During EEN (T1)** | | **End EEN (T2)** | | **Follow-up (T3)** | |
|  | **Responders**  **(n=10) vs**  **Non-responders (n=12)** | **P**^a^ | **Responders**  **(n=10) vs**  **Non-responders (n==11)** | **p^a^** | **Responders**  **(n=7) vs**  **Non-responders (n=11)** | **p^a^** | **Responders**  **(n=7) vs**  **Non-responders (n=10)** | **p^a^** |
| Total bile acid concentration *(mM, median (IQR))* | 0.51 (0.23-1.08) vs 0.57 (0.37-1.24) | 0.8229 | 0.88 (0.71-1.22) vs 1.63 (0.38-2.42) | 0.5824 | 0.53 (0.28-2.12) vs 0.78 (0.50-2.92) | 0.1743 | 1.77 (0.66-2.93) vs  1.77 (0.46-4.03) | 0.8868 |
| Bile acid hydrophobicity index *(median (IQR))* | 0.70 (0.67-0.72) vs 0.54 (0.27-0.76) | 0.1802 | 0.71 (0.64-0.73) vs 0.68 (0.44-0.73) | 0.4262 | 0.69 (0.63-0.77) vs  0.75 (0.73-0.79) | 0.3651 | 0.72 (0.45-0.82) vs  0.67 (0.59-0.76) | 0.7396 |
| % secondary bile acids *(median (IQR))* | 92.2% (79.6-94.9)vs  72.9% (27.2-91.9) | 0.1229 | 92.7% (89.4-97.2) vs 84.1% (0.60-94.7) | 0.1517 | 94.5% (90.4-98.3) vs  91.2% (85.0-93.8) | 0.1743 | 92.9% (76.4-96.6) vs  86.3% (29.1-90.2) | 0.1932 |
| Bile acid concentrations are expressed in mM and were measured using HPLC. No significant differences in bile acid concentrations or compositions were seen between responders and non-responders. IQR: inter quartile range, mM, millimol ***^a^*** *Mann-Whitney U test* | | | | | | | | |

| **Supplementary Table 12 │**  Fecal bile acid composition in healthy controls versus responders and non-responders at T3. | | | | | |
| --- | --- | --- | --- | --- | --- |
|  | **Healthy controls (n=32)** | **Responders (T3) (n=10)** | **Non-responders (T3) (n=7)** | **p^a^** | **Post-hoc test^b^** |
| Total bile acid concentration *(mM, median (IQR))* | 0.84 (0.42−1.42) | 1.77 (0.66-2.93) | 1.77 (0.46-4.03) | 0.0417 | HC vs R: no  HC vs NR: no |
| Bile acid hydrophobicity index *(median (IQR))* | 0.79 (0.73−0.81) | 0.72 (0.45-0.82) | 0.67 (0.59-0.76) | 0.0204 | HC vs R: no  HC vs NR: yes |
| % secondary bile acids *(median (IQR))* | 93.7% (85.3−96.3) | 92.9% (76.4-96.6) | 86.3% (29.1-90.2) | 0.0851 | HC vs R: no  HC vs NR: no |
| Bile acid concentrations are expressed in mM and were measured using HPLC. At T3, concentrations and compositions were not different between controls and responders whereas there was a difference in bile acid hydrophobicity between non-responders and controls. IQR: inter quartile range.**^a^** Kruskall-Wallis test  **^b^** Dunn’s multiple comparison post-hoc test. | | | | | |
