# Supplementary figures and images for: Exclusive enteral nutrition mediates gut microbial and metabolic changes that are associated with remission in children with Crohn’s disease
Source: Sci Rep. 2020 Nov 3;10:18879. doi: 10.1038/s41598-020-75306-z (PMC7609694; doi:10.1038/s41598-020-75306-z)

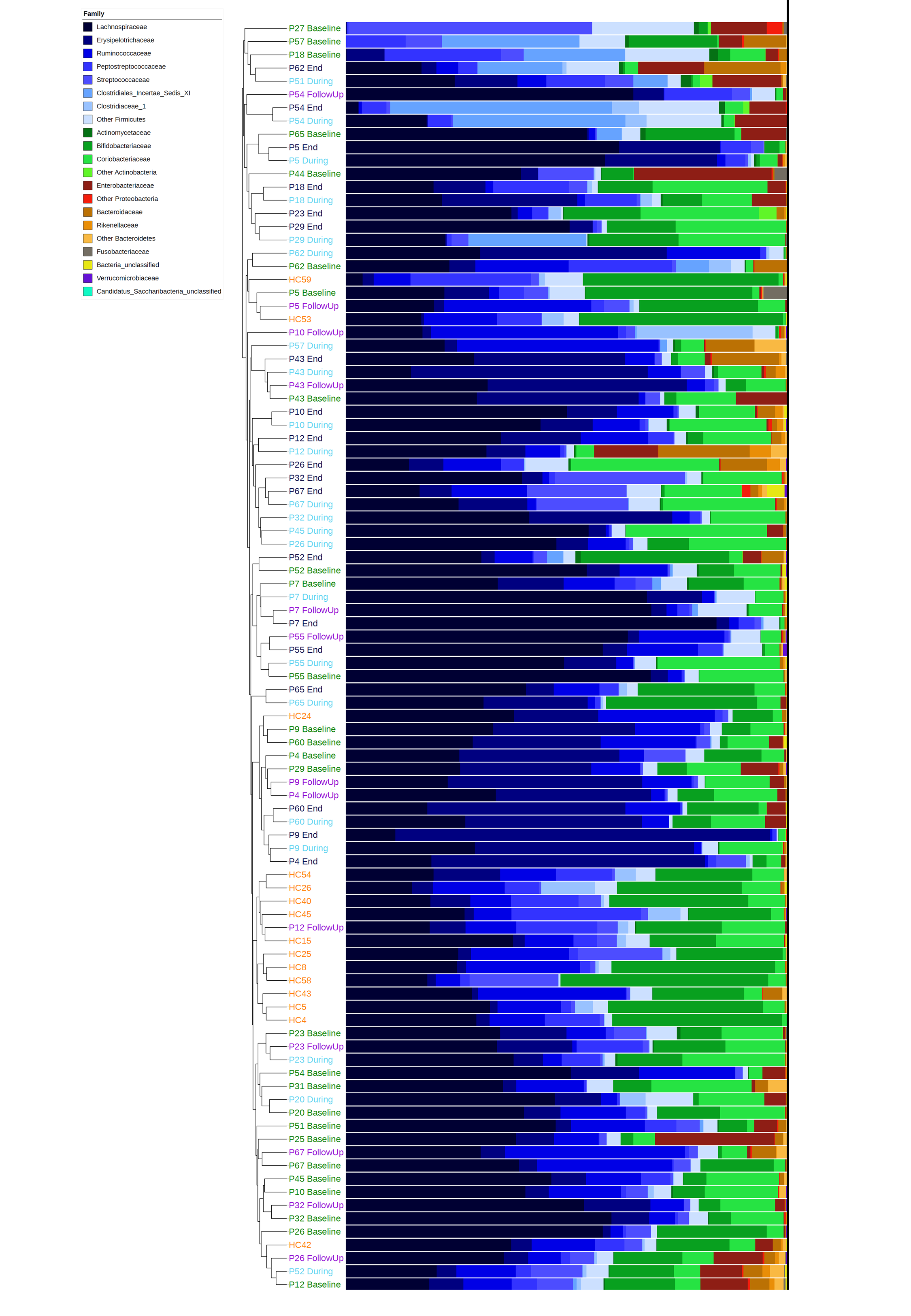

Supplement: Supplementary file 3 — Supplementary Figure 1. [file 41598_2020_75306_MOESM3_ESM.tif]

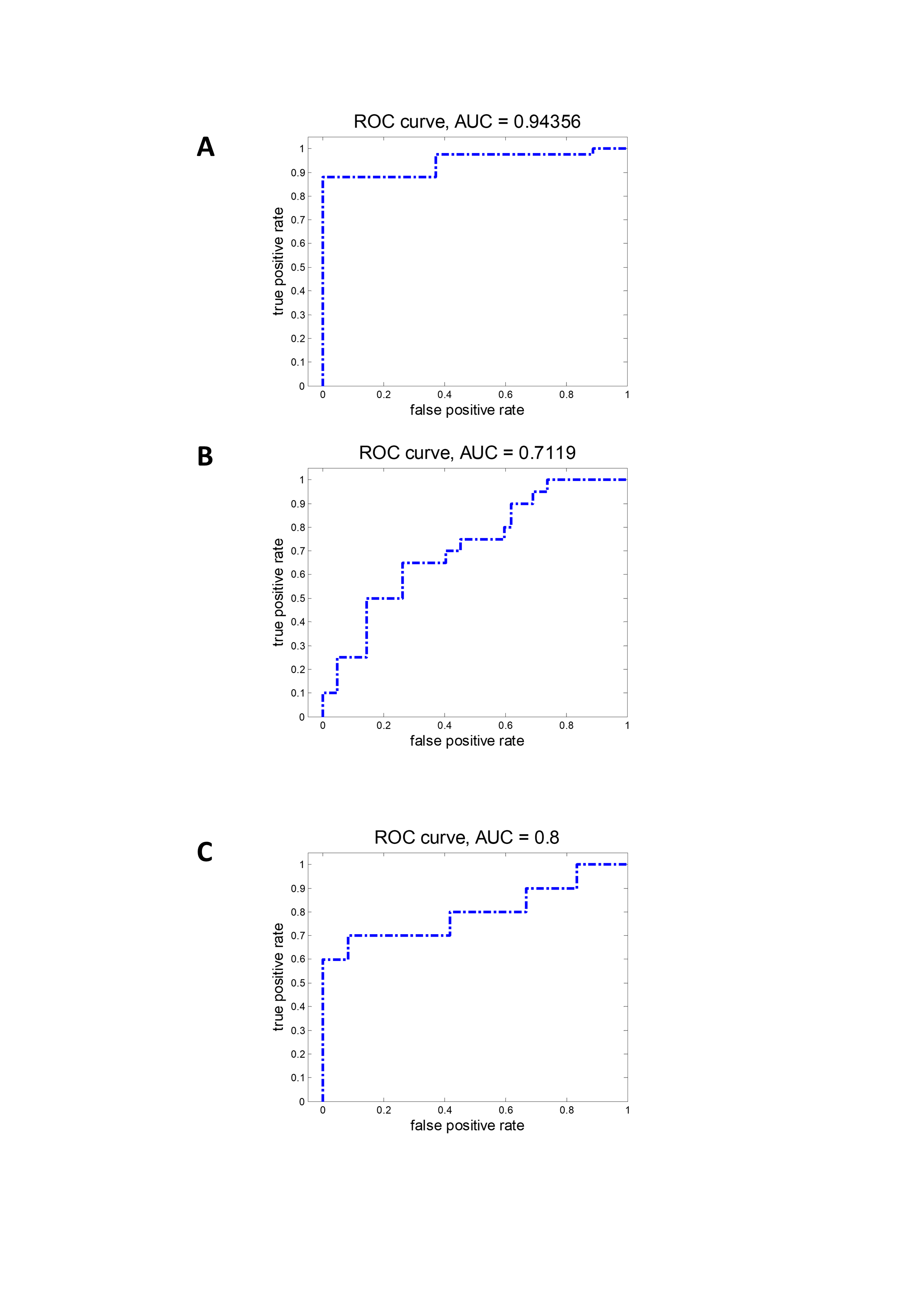

Supplement: Supplementary file 4 — Supplementary Figure 2. [file 41598_2020_75306_MOESM4_ESM.tif]

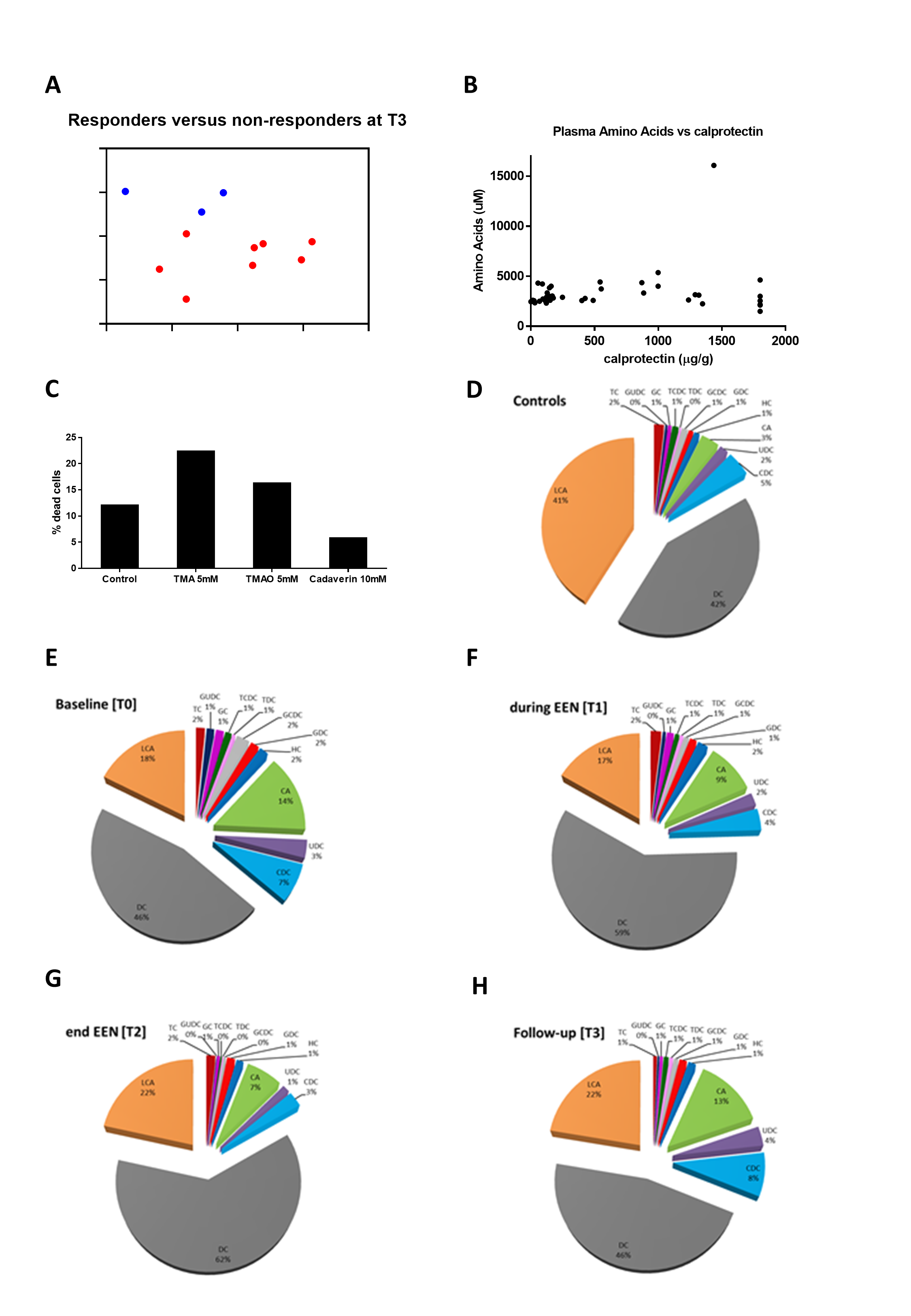

Supplement: Supplementary file 5 — Supplementary Figure 3. [file 41598_2020_75306_MOESM5_ESM.tif]
